# It’s Tea Time: Interference of Ayahuasca Brew on Discriminative Learning in Zebrafish

**DOI:** 10.3389/fnbeh.2018.00190

**Published:** 2018-08-27

**Authors:** Bruno Lobao-Soares, Paulianny Eduardo-da-Silva, Hugo Amarilha, Jaquelinne Pinheiro-da-Silva, Priscila F. Silva, Ana Carolina Luchiari

**Affiliations:** ^1^Departamento de Biofísica e Farmacologia, Centro de Biociências, Universidade Federal do Rio Grande do Norte, Natal, Brazil; ^2^Departamento de Fisiologia, Centro de Biociências, Universidade Federal do Rio Grande do Norte, Natal, Brazil

**Keywords:** Santo Daime, *Danio rerio*, memory, β-carbolines, DMT, object discrimination task

## Abstract

Ayahuasca is a psychoactive brew traditionally used in shamanistic and vegetalistic rituals and has recently received lot of attention due to potential cognitive benefits. Ayahuasca effects are caused by the synergistic interaction of β-carbolines (harmine, harmaline and tetrahydroarmine) contained in *Banisteriopsis caapi* stalks combined with the N,N-dimethyltryptamine (DMT) from *Psychotria viridis* leaves, a potent agonist to serotonin (5-HT) receptors. The present study approaches the effects of chronic and acute exposure to two Ayahuasca concentrations (0.1 and 0.5 ml/L) on the cognitive ability to discriminate objects in a one-trial learning task in zebrafish. Based on the combination of concentrations and exposure regimens, we divided adult zebrafish in five treatment groups: acute 0.1 and 0.5 ml/L, chronic 0.1 and 0.5 ml/L, and control 0.0 (*n* = 20 for each group). Then we tested them in a memory task of object discrimination. Acute Ayahuasca exposed groups performed similarly to the control group, however chronically treated fish (13 days) presented both impaired discriminative performance and locomotor alterations. Overall, these results indicate that Ayahuasca is a potent psychoactive drug that, in chronic exposure, negatively affects mnemonic parameters in zebrafish. In single exposure it does not affects cognitive performance, but the higher concentration (0.5) affected locomotion. Moreover, we reinforce the importance of the zebrafish for behavioral pharmacological studies of drug screening, in special to psychedelic drug research.

## Introduction

Learning is a critical ability for an individual’s development and fitness because it allows for gathering information, processing, and changing behavior to properly respond to several different situations. A complementary process to learning is memory, which confers the ability to create representations of experiences and information that can persist for different periods of time (Vishnoi et al., 2016). Both learning and memory are dependent on neuronal plasticity (Kolb and Whishaw, 1998; Saar et al., 2002; Ghosh et al., 2016), a very delicate process that is affected by psychoactive drugs (Gould, 2010). Drugs interfere on the learning and memory processes by modifying attention, perceptual, or motivational processes, which directly affect the formation of prominent traces (Vik et al., 2004; Kuypers and Ramaekers, 2007; Potvin et al., 2018).

Notwithstanding, some drugs are claimed to improve cognition. Among them, Ayahuasca, a hallucinogen beverage prepared through the decoction of *Banisteriopsis caapi* stalks and *Psychotria viridis* leaves, has gained ground. Regular users declare a number of benefits obtained from the brew intake, such as mind healing, increased self-knowledge, improved memory and persistently elevated mood (Frecska et al., 2012, 2016). Ayahuasca’s effects are derived from the combined action of β-carbolines (harmaline, harmine and tetrahydroharmine) which act as reversible monoamine oxidase inhibitor (MAOI), with N,N-dimethyltryptamine (DMT), an indole alkaloid similar to serotonin (5-HT). The inhibition process of the MAO caused by the β-carbolines results in a higher oral bioavailability of DMT, which acts mainly as an agonist of the 5-HT receptors (Fuller et al., 1970; Yasuhara et al., 1972; McKenna et al., 1984; McKenna, 2004). As a result, DMT promotes similar effects as the 5-HT itself (Rabin et al., 2002) and together with MAOI leads to increased BDNF levels in humans (dos Santos and Hallak, 2017), which seems to be related to amelioration of some psychological disease symptoms such as anxiety, obsessive-compulsive behavior and depression (Bouso et al., 2012; Cai et al., 2015).

However, Ayahuasca use is still controversial. Studies applying Ayahuasca to animal models have argued that chronic exposure may provoke toxic and cognitive-impairment effects, such as neuronal loss (Figueroa, 2012; Pic-Taylor et al., 2015) and increased serotonergic activity that induces neurodegeneration (Jiang et al., 2015). The zebrafish (*Danio rerio*) has attracted scientific attention in recent years, and is at the forefront of developmental biology studies (Grunwald and Eisen, 2002; Kalueff et al., 2014), in addition to being a promising model in predictive validation for drug research (Gerlai et al., 2000; Rico et al., 2007; Luchiari and Chacon, 2013; Chacon and Luchiari, 2014; Sterling et al., 2015; Santos et al., 2016). Both adult and larval zebrafish have been applied in studies approaching basic neuroscience questions and it has been successfully used to understand brain development and functioning in humans due to the high conservation of many molecular pathways, genes and protein products with mammals (Holzschuh et al., 2001; Kaslin and Panula, 2001; Kaslin et al., 2004; McLean and Fetcho, 2004; Faraco et al., 2006; Prober et al., 2006). Although the zebrafish has been used as one of the best animal models for high throughput screening in neuroscience, much less is known about the learning and memory processes in adult fish.

In a previous study using zebrafish, we have reported that anxiolytic effects are dependent on the concentration used, and slightly higher concentration can cause large suppressive effects on locomotion (Savoldi et al., 2017). In this sense and due to the widespread use of Ayahuasca worldwide (about 20,000 Ayahuasca users in the world), studies on the effects of this drug on cognitive processing are urgent. Therefore, this study evaluates the effect of Ayahuasca exposure, both acute and chronic, in the performance of zebrafish on a cognitive task. For this, we used a more vulnerable type of memory, based on one-trial recognition test (Oliveira et al., 2015), due to its sensibility to most psychostimulant substances and rapid response for highly impacting psychedelic substances such as Ayahuasca.

## Materials and Methods

### Animals and Housing

In the present study 100 adult zebrafish (*Danio rerio*, wild type, both sexes, 2.5 ± 0.2 g) were used. Fish stock was acquired from a local farm in Natal (Brazil) and transferred to the vivarium of the Fish Laboratory (Physiology Department—UFRN) 60 days previous to the tests. Fish were held in communal tanks (50 L, 100 × 30 × 50 cm, width × depth × height) connected through a recirculation system with multistage filtration, including a mechanical filter, biological filter, activated carbon filter and UV light-sterilizing unit. Temperature, pH and oxygen were measured daily (average values: 28°C, pH 6.7, O_2_ 6 mg/L) and the photoperiod set at 12:12 light:dark cycle. Fish were fed twice a day with commercial food (Nutricom Pet: 38% protein and 4% lipids), and frozen *Artemia salina*. All procedures for the present study were approved by the Ethical Committee for Animal Use of the Federal University of Rio Grande do Norte (CEUA n° 053/2016).

### Ayahuasca Exposure

Ayahuasca brew was obtained from the religious group “Igreja da Barquinha,” Ji-Paraná, Rondonia State (Brazil). The preparation followed the traditional recipe. Briefly, 50% of *Banisteriopsis caapi* and 50% of *Psychotria viridis* leaves are boiled to concentration into water for several hours, infused water is cooled down and transferred to bottles to be stored in a refrigerator. The infusion used in the present study has been previously analyzed by gaseous chromatography and presented 0.36 ± 0.01 mg/ml of DMT, 1.86 ± 0.11 mg/ml of harmine, 0.24 ± 0.03 mg/ml of harmaline and 1.20 ± 0.05 mg/ml of tetrahydroharmine (Savoldi et al., 2017).

After the acclimating period in the vivarium, fish were separated into five groups (*n* = 20 each) and held in glass tanks (40 × 25 × 20 cm), composing the groups for Ayahuasca exposure before the one-trial recognition test. Tanks were maintained with aeration given by an air stone and the water volume was exchanged in 30% daily to keep water quality.

**Figure 1:**
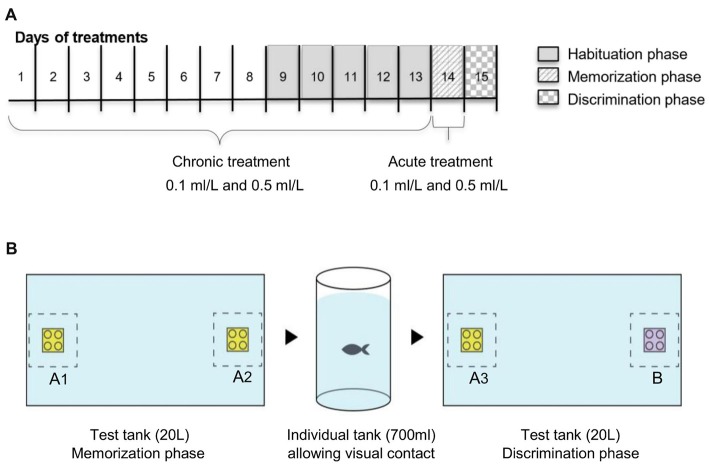
Schematic representation of the experimental design: **(A)** timeline for Ayahuasca exposure and **(B)** one-trial discrimination test. Along the line, the 15 days of experiment. Five groups of Ayahuasca exposure were tested: chronic 0.1 ml/L, chronic 0.5 ml/L, acute 0.1 ml/L, acute 0.5 ml/L and 0.0 ml/L (control). One-trial discrimination test was split into three phases: 5 days of tank habituation, memorization phase (with two identical 3D objects—A_1_ × A_2_) and discrimination phase (with two different 3D objects—A_3_ × B).

For the drug exposure, we used two exposure regimes, acute and chronic and two doses, 0.1 ml/L and 0.5 ml/L of Ayahuasca. We chose the doses based on our previous work which analyzed four acute doses (0.1 ml/L, 0.5 ml/L, 1 ml/L and 3 ml/L; Savoldi et al., 2017). Acute 1 and 3 ml/L decrease locomotion and increase anxiety impairing fish behavior, so we exclude these higher doses from the present experiment. The combination of doses and exposure regime create five treatments: chronic 0.1 ml/L (C0.1), chronic 0.5 ml/L (C0.5), acute 0.1 ml/L (A0.1), acute 0.5 ml/L (A0.5), and the control 0.0 ml/L (0.0). For the chronic groups, fish were daily transferred (gently using a net) to a small tank (2 L) containing the Ayahuasca concentration for 60 min exposure, and then transferred back to the holding tank. This procedure was repeated 13 consecutive days (Figure 1). For the acute treatment, fish were daily transferred to a small tank (2 L) containing only water, where it was kept for 60 min along 13 days, and on the 14th day fish were exposed to 0.1 ml/L or 0.5 ml/L Ayahuasca for 60 min previous to the one-trial recognition test (Figure 1). The control group was handled everyday as the other groups but never exposed to Ayahuasca. Ayahuasca concentration for fish exposure was prepared fresh daily and discarded immediately after. Concentrations used were based on previous results using the same brew (Savoldi et al., 2017).

### One-Trial Discrimination Test

The discrimination test was divided into three phases: Habituation (1); Memorization (2); and Discrimination (3; Figure 1B). The Habituation phase (1) consisted of the presentation of the test tank to the animals with the goal of diminishing novelty and isolation stress. This phase took 5 days and fish from each Ayahuasca group were allowed to explore the tank used for the test (glass tank, 40 × 25 × 20 cm, all covered in white). On the first habituation day, 10 fish from each Ayahuasca group were put together into the tank for 20 min. On the following days, the number of animals was progressively decreased (five fish on the 2nd, 3–4 fish on the 3rd, two fish on the 4th and one fish on the last day), so that on the last day of this phase fish could explore the tank alone for 20 min every time.

On the day after, the Memorization phase (2) took place. Each fish was allowed to explore the tank for 20 min, but this time there were two identical 3D objects (Lego^®^ blocks, same size, color and shape, named A_1_ and A_2_) displayed in opposite sides beside the two smallest walls of the tank. On the memorization phase, fish from the acute Ayahuasca groups were exposed to 0.1 ml/L or 0.5 ml/L (2 L tank) during 60 min before being placed into the test tank. Fish from the chronic Ayahuasca and the control groups were placed for 60 min in small tank (2 L) containing clear water before going to the test tank. Fish behavior in the test tank was recorded using a video camera (Sony Digital Video Camera Recorder; DCR-SX45) placed 1 m above the tank. At the end of 20-min exploring the tank with objects, each fish was gently removed to its holding tank, separated from each other by a transparent partition to maintain fish identity but allowing visual contact.

On the next day, the Discrimination phase (3) went on. For this, one of the objects previously used was replaced by a new one, which had similar size and shape, but different color (objects A_3_ and B). The novel object (B) were always randomized between the animals. Objects in colors green and blue were not used during this trials due to previous records of zebrafish preference for these colors (Avdesh et al., 2010; Oliveira et al., 2015). On this phase, each fish was transferred to the test tank for 20 min exploring the objects and behavior was recorded from above. There was no manipulation or drug exposure going on before the test. All the transferences of fish between their residence tanks and test tanks occurred gently and rapidly with a net.

### Behavioral and Data Analysis

The behavior recorded in video was tracked through the software ZebTrack (v2.3; Pinheiro-da-Silva et al., 2017) on the MATLAB platform (R2013A 8.1.0.604). The parameters analyzed were: average swimming speed, maximum swimming speed, total distance traveled, freezing and time exploring each object. We considered the animal’s residence time in a 3-cm area around the objects to define exploration behavior (Lucon-Xiccato and Dadda, 2014; Pinheiro-da-Silva et al., 2017).

To apply statistical inference, data were evaluated through exploration analysis due to possible issues with outliers, homoscedasticity, normality, inflated zero, collinearity and independent variables as suggested by Zuur et al. (2010). The difference of exploration time between objects on each phase was determined by Bayesian inference, an analysis that allows for complete posterior distribution to statistical models, creating richer inferences than more traditional hypothesis tests (Kruschke et al., 2012). Bayesian analyses use a greater trust interval (highest density interval—HDI) instead of the trust interval applied by frequency analyses. The HDI reduces uncertainties, indicating values with greater credibility and covering 95% of the data distribution. For our data, the comparison value was set at about 0, and the Region of Practical Equivalence—ROPE was defined at ±5 (Pinheiro-da-Silva et al., 2017).

An exploration index was calculated to evaluate differences in exploration on each phase of the test, according to Akkerman et al. (2012) and May et al. (2016). The exploration index calculated for the memorization phase considered the total time exploring object A1 and A2 (E_m_ = A_1_ + A_2_). The discrimination index for the discrimination phase considered the difference between the exploration of the known object and the new object (D_i_ = B − A_3_). Linear regression analysis was performed to determine whether E_m_ was predictive of D_i_, that is, whether time exploring on the memorization phase could be related to the performance in discriminating the new object on the discrimination phase. The discrimination indices were then compared by Bayesian analysis for each group, considering the theoretical mean as 0 (we used R program to run Bayesian analysis; Team, 2015).

The locomotor parameters registered on the memorization and discrimination phases were compared by One-Way ANOVA, followed by Tukey test, in which *p* value was considered significant when below 0.05 (we used Statistic 8.0 Stat Soft to run ANOVA analysis).

## Results

**Figure 2:**
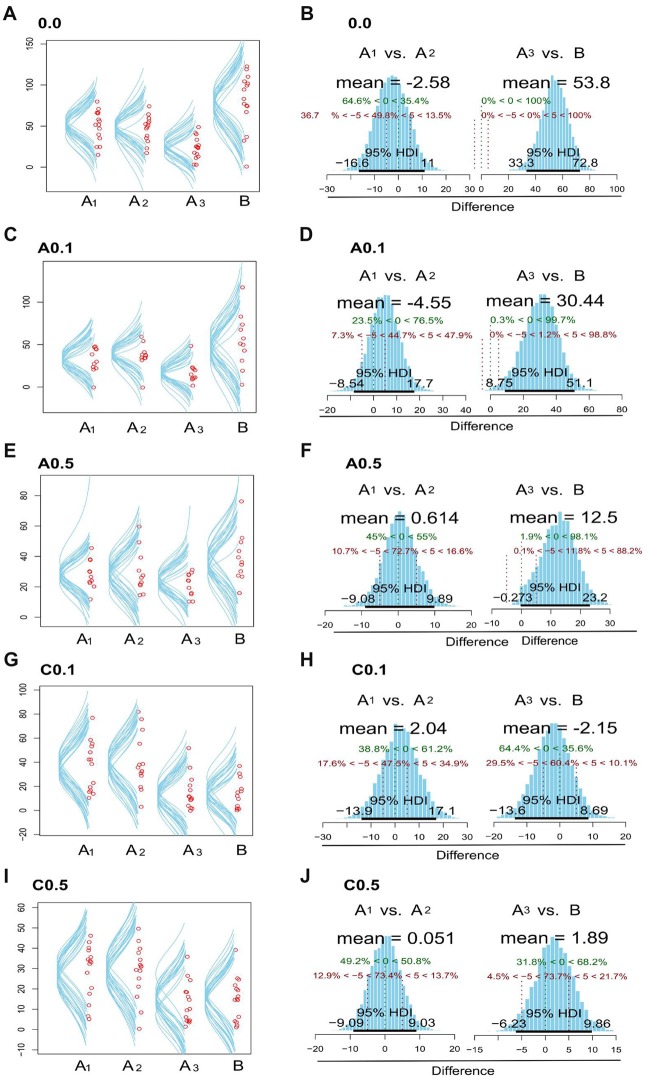
Plots zebrafish exploration of objects A_1,_ A_2,_ A_3_ and B. Fish were observed for 15 min and the time spent near the objects were considered as exploration. The left side column **(A,C,E,G,I)** shows the dispersion of median values for exploration time in each object, both in memorization and in discrimination phases, for the 0.0, A0.1, A0.5, C0.1 and C0.5 groups. The right column **(B,D,F,H,J)** exhibits posterior distribution of the Bayesian analysis: comparison between the exploration time of objects A_1_ vs. A_2_ and A_3_ vs. B for the five groups tested. Green dashed lines (central dashed line) indicate the comparison value = 0 and red dashed lines (lateral dashed lines) indicate the Region of Practical Equivalence (ROPE) ±5. 95% highest density interval (HDI) is marked by the black bar on the floor of the distribution. For further details and statistical analyses, see the “Results” section.

Figure 2 presents data with posterior predictive distribution of exploration time of the objects on both phases of the test for groups 0.0, A0.1, A0.5, C0.1 and C0.5, and Bayesian analysis between phases for all groups. The predictive distribution for these data show that groups 0.0, A0.1 and A0.5 had no relevant difference between the exploration time on each phase (Mean Difference = 0.0: −1.57, 95% HDI = [−18.8, 13.9]; A0.1: 0.60, 95% HDI = [−15.1, 15.4]; A0.5: −1.21, 95% HDI = [−9.44, 7.41]; C0.1: 24.7, 95% HDI = [15.3, 4.8]; C0.5: 14.8, 95% HDI = [8.53, 21]). In contrast, the groups C0.1 and C0.5 showed a reduction in the object exploration time on the discrimination phase (Figures 2A,C,E,G,I).

Then, we compared the exploration time of each object in both phases of the test (Memorization: A_1_ × A_2_ and Discrimination: A_3_ × B). The control group (0.0) presents no difference on exploration time of the objects on the memorization phase (Mean Difference = A_1_ × A_2_ = −2.58, 95% HDI = [−16.2, 11]). However, there was clear difference on exploration time on the discrimination phase (Mean Difference = A_3_ × B = 53.8, 95% HDI = [33.3, 72.8]; Figure 2B). The group A0.1 presented no difference on the object exploration time during the memorization phase (Mean Difference = −4.55, 95% HDI = [−8.54, 17.7]), but presented relevant difference on the exploration time on the discrimination phase (Mean Difference = 30.4, 95% HDI = [8.75, 51.1]; Figure 2D). The group A0.5 showed no difference on the exploration time of A_1_ × A_2_ objects (Mean Difference = 0.614, 95% HDI = [−9.08, 9.89]), but Bayesian inference showed a trend towards increasing exploration time of object B on discrimination phase (Mean Difference = 12.5, 95% HDI = [−0.27, 23.2]), although 12% of the data distribution is on the practical equivalence zone (−0.5–0.5; Figure 2F). On the contrary, groups C0.1 and C0.5 did not present any difference on the objects exploration time both on the exploration and the discrimination phases (C0.1: Mean Difference = A_1_ × A_2_ = 2.04, 95% HDI = [−13.9, 17.1], A_3_ × B = −2.15, 95% HDI = [−13.6, 8.69]; C0.5: Mean Difference = A_1_ × A_2_ = 0.05, 95% HDI = [−9.09, 9.03], A_3_ × B = 1.89, 95% HDI = [−6.23, 9.86]; Figures 2H,J, respectively).

**Figure 3:**
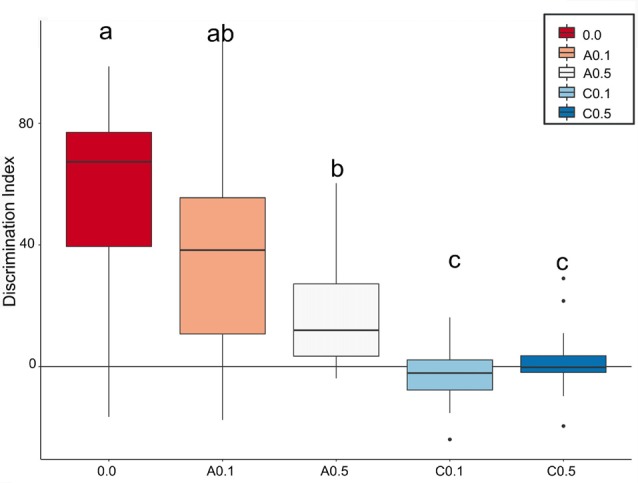
Index box plots represent the relative median values of discrimination index (D_i_ = B − A3) for the five groups tested in comparison with a theoretical mean of 0. Groups C0.1 and C0.5 together showed the lowest values of discrimination, while the A0.1 presented discrimination similar to control group. Different letters represent statistic difference between groups. Isolated dots correspond to outliers’ values. For further details and statistical analyses, see the “Statistical Analysis” section.

Based on how much the animals explored each object, a discrimination index (D_i_) was calculated. Figure 3 depicts the box plots of the median of D_i_ for the 5 experimental groups. The closer the median value to zero, the lower the object discrimination. The Bayesian analyses carried out for paired comparison between groups showed relevant differences between groups 0.0 vs. A0.5 (Mean Difference = 40.4, 95% HDI = [15.9, 64.8]), 0.0 vs. C0.1 (Mean Difference = 59.5, 95% HDI = [38.8, 80.7]), 0.0 vs. C0.5 (Mean Difference = 55.4, 95% HDI = [35.6, 78.2]), A0.1 vs. C0.1 (Mean Difference = 39.6, 95% HDI = [12.5, 64.9]), A0.1 vs. C0.5 (Mean Difference = 34.6, 95% HDI = [9.18, 61.0]), A0.5 vs. C0.1 (Mean Difference = 19.5, 95% HDI = [3.91, 34.7]), and A0.5 vs. C0.5 (Mean Difference = 14.6, 95% HDI = [0.01, 30.7]). However, the analyses presented no significant differences between groups 0.0 vs. A0.1 (Mean Difference = 25.2, 95% HDI = [−10.9, 53.8]), A0.1 vs. A0.5 (Mean Difference = 19.5, 95% HDI = [−9.3, 49.1]) and C0.1 vs. C0.5 (Mean Difference = −4.04, 95% HDI = [−12.8, 4.88]). All Bayesian comparison are presented in Supplementary Figure S1.

**Figure 4:**
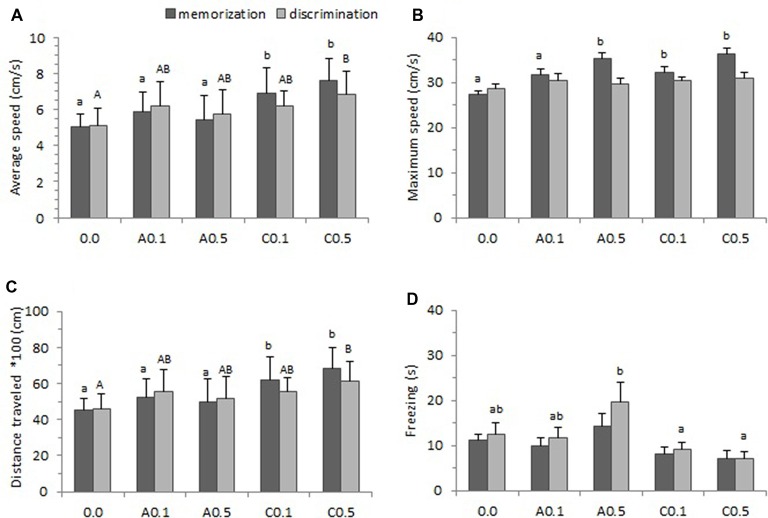
Locomotor parameters observed during one-trial learning test. **(A)** Average speed, **(B)** Maximum speed, **(C)** Total distance traveled and **(D)** Freezing. Five groups of Ayahuasca exposure were tested: 0.0 ml/L (control: 0.0, *n* = 20), acute 0.1 ml/L (A0.1, *n* = 20), acute 0.5 ml/L (A0.5, *n* = 20), chronic 0.1 ml/L (C0.1, *n* = 20), and chronic 0.5 ml/L (C0.5, *n* = 20). Fish behavior was recorded during 15 min on the memorization phase and 15 min during the discrimination phase. One-way ANOVA was applied for comparisons between groups on the memorization and on the discrimination phase. At least one different letter or *indicates statistical significance at *p* < 0.05. Lower case letters were used for the memorization phase and upper case letter for the discrimination phase. For details see “Results” section.

Figure 4 presents the locomotor parameters (average speed, maximum speed, total distance traveled and freezing) of zebrafish exposed to Ayahuasca on the memorization and discrimination phases of the one-trial discrimination test. A one-way ANOVA found statistical difference for average swimming speed between groups both on the memorization phase (*F*_(4,74)_ = 12.74, *p* < 0.001) and on the discrimination phase (*F*_(4,74)_ = 4.61, *p* = 0.002). The Tukey’s test is a *post hoc* that applies simultaneously to the set of all pairwise comparisons, and here it showed that groups C0.1 and C0.5 presented higher average speed than the other groups on the memorization phase (*p* < 0.05), and that group C0.5 showed the highest average speed on the discrimination phase (*p* < 0.05; Figure 4A).

We analyzed the maximum speed to verify if there were alterations in the animal’s standard locomotor characteristics due to the substance exposure. A one-way ANOVA presented statistical significance between groups on the memorization phase (*F*_(4,74)_ = 8.71, *p* < 0.001) but the effect was found non-significant on the discrimination phase (*F*_(4,73)_ = 0.93, *p* = 0.44). Tukey’s *post hoc* test indicated that, on memorization phase, the groups A0.5, C0.1 and C0.5 presented higher maximum speed than other groups (*p* < 0.05; Figure 4B).

The one-way ANOVA performed for all groups, revealed significant effect on total distance traveled during the test both on memorization phase (*F*_(4,73)_ = 12.19, *p* < 0.001) and discrimination phase (*F*_(4,73)_ = 4.52, *p* = 0.003). Tukey’s *post hoc* test showed that, on the memorization phase, animals from groups C0.1 and C0.5 traveled more distance during the test than those of other groups (*p* < 0.05). Yet on the discrimination phase only the group C0.5 presented higher distance traveled (*p* < 0.05; Figure 4C).

Freezing behavior is a total absence of movement and may indicate higher level of anxiety in the animal. In this study, a one-way ANOVA applied for all groups showed no significant effect for the memorization phase (*F*_(4,73)_ = 2.32, *p* = 0.06) but found effect significant on the discrimination phase (*F*_(4,73)_ = 3.65, *p* = 0.009). Tukey’s *post hoc* test revealed that groups A0.5 remained longer in freezing than C0.1 and C0.5 groups on the discrimination phase (*p* < 0.05; Figure 4D).

## Discussion

In this study, we present the effects of Ayahuasca on a very sensible and disruptive (labile) type of memory that is one-trial object discrimination. We confirm the zebrafish ability to perform objects memorization and discrimination, however our results provide evidence that while acute exposure to the 0.1 ml/L and 0.5 ml/L Ayahuasca does not alter object memorization and discrimination, the same dosages on chronic exposure impair discriminative learning, observed by the decrease on the exploration time as well as on the discrimination index.

Zebrafish is by nature a highly explorative and novelty attracted animal (Sison and Gerlai, 2010; Luchiari et al., 2015) that uses information obtained from the environment to learn about it. In this view, our data corroborate other studies showing the zebrafish ability to learn about objects and use the previous acquired information to discriminate a novel environmental feature (Oliveira et al., 2015; Santos et al., 2016; Pinheiro-da-Silva et al., 2017). The ability to learn and change behavior according to one’s experiences is highly valuable to all animal species, as it provides several benefits to fitness. Such ability is only possible due to the modulatory characteristic of the nervous system, particularly the hippocampus in mammals (Mumby et al., 2002; Good et al., 2007; Barker and Warburton, 2011) or homologous areas in other species—in fish represented by the lateral pallium (Rodríguez et al., 2002). However, it is exactly this specific part of the whole nervous system (the hippocampus analogous areas) that is specially affected by psychoactive drugs (White et al., 2013; Davidson et al., 2018), such as Ayahuasca. Nevertheless, the cognitive behaviors measured here are limited and future essays should be applied to verify in-depth cognitive effects.

Currently, there are no pharmacological studies in the literature reporting the Ayahuasca effects on zebrafish learning and memory (or any other fish species). The structural homology and genetic similarity between mammals and zebrafish offers an opportunity to understand Ayahuasca mechanisms of action with translational view. Indeed, zebrafish is a reliable experimental model for the screening of psychoactive, anti-depressive/anxiolytic and other drugs (Stewart et al., 2014).

Regarding the psychoactive drug approached in this study, Ayahuasca affects one’s brain because of the abundance of β-carbolines and DMT available in it (McKenna et al., 1984). The β-carbolines inhibit the action of MAO and thus, allows the great bioavailability of DMT. This molecule (DMT) would then act mostly as a potent serotoninergic agonist with its main action on 5-HT2A receptors, but also acting on 5-HT_1A_, 5-HT_1B_, 5-HT_1D_, 5-HT_2B_, 5-HT_2C_, 5-HT_5A_, 5-HT_6_ and 5-HT_7_ receptors (Carbonaro and Gatch, 2016). Indirectly, the β-carbolines also act as serotoninergic, dopaminergic and noradrenergic agonists, for MAO possess action on the degradation of all of those monoaminergic neurotransmitters (McKenna, 2004; Herculano and Maximino, 2014).

In this study, zebrafish exposed to acute 0.1 ml/L and 0.5 ml/L Ayahuasca showed discriminative performance comparable to the control group (Figures 2, 3). Moreover, these two concentrations of Ayahuasca did not trigger locomotor alterations (Figure 4). Zebrafish were exposed to Ayahuasca 60 min before the memorization phase of the task, thus fish were under the action of the drug during the most important part of the memory test: perceiving, attending and exploring the objects present in the tank. As shown by Savoldi et al. (2017), low doses of Ayahuasca seems to decrease anxiety-like behavior in zebrafish, allowing the animals to better explore and learn about the environment.

Despite the actions of DMT on the 5-HT receptors, it has also been reported to increase the concentrations of dopamine and norepinephrine in the amygdala and hippocampus (de Castro-Neto et al., 2013). Therefore, it seems that a low dose of Ayahuasca offers the ideal amount of brain stimulation to confer decreased anxiety and increased overall perception of the situation to obtain as much information as possible. Although the low doses of Ayahuasca did not cause any amelioration for the zebrafish object discrimination (results were comparable to control), the present task counts simply on one-trial exposure to access learning and memory. In this sense, future studies approaching more complex tasks such as associative conditioning or time and place learning would be valuable to compose a more complete picture of the low Ayahuasca doses on the cognitive function.

On the other hand, zebrafish exposed to 0.1 ml/L and 0.5 ml/L Ayahuasca for 13-days (chronic) previous to the cognitive task employed in this study presented impaired ability to discriminate objects. Additionally, these groups (C0.1 and C0.5) presented increased swimming speed and distance traveled compared to the other concentrations. Although these fish have shown higher activity, the objects exploration on the memorization phase was significantly low (Figure 2). The increased overall activity together with decreased attention and exploration behavior of chronically treated animals suggest signals related to anxiety. It is known that hyperactivity and anxiety-like behavior are highly correlated in zebrafish (Kalueff et al., 2013), which seems to be the case of the chronic Ayahuasca groups. The continued combination of DMT and harmaline induces to excessive activation of the serotonergic system, possibly leading to a state of 5-HT toxicity (Pic-Taylor et al., 2015).

With exception of the 5-HT5, all the 5-HT receptors subtypes can be potentially modulated by DMT and are reported in brain regions related to learning and memory (Meneses, 1999). Studies have shown that agonistic activity at 5-HT2_A_ receptor improves memory (Alhaider et al., 1993; Harvey, 2003). DMT is described as having its most evident pharmacological activity as a 5-HT2_A_ agonist, therefore an increase in mnemonic capacity could be expected in the tested protocol. Nevertheless, in the present study the long-term exposure to Ayahuasca seems to suppress this benefit. For instance, in rats, excessive activation of 5-HT2_A_ receptor impairs latent inhibition (Cassaday et al., 1993). We could consider that 5-HT, as previously reported for dopamine (França et al., 2015), might exert modulation on learning and memory in an U inverted shape, i.e., to achieve an optimal performance, an intermediate level of serotonergic system activity should be preferable. However, further studies are necessary for understanding this possible mechanism and also the role of the other 5-HT receptors related to DMT effects on learning and memory processes. In addition, also the dopaminergic and noradrenergic function may be modulated by beta-carbolines in ayahuasca tea. Indeed, these two neurotransmitters also have been described as critical modulators of learning processes (França et al., 2015; Atucha et al., 2017).

Furthermore, 5-HT can cross-regulate acetylcholine release in the brain (Jackson et al., 1988; Jäkälä et al., 1992; Sparks et al., 2018) and harmaline also binds to other neurotransmitter receptors (norepinephrine, dopamine, glutamate receptors; Glennon et al., 2000; Miwa et al., 2000; Grella et al., 2003; Beitz and Saxon, 2004; Kralic et al., 2005; Paterson et al., 2009) This whole excessive alteration of the neurotransmitter systems may be related in the present data to decreased attention and learning observed after chronic Ayahuasca exposure. In fact, both chronic groups C0.1 and C0.5 presented reduced exploration time, and discrimination index significantly inferior to the control and acute groups. In other words, those groups did not replicate the typical behaviors observed on the control group. Currently, the use of microdoses (small doses without toxic effects named subclinical doses, such as 0.1) of psychedelics has gained popularity due to the positive consequences described on mood and cognition (Johnstad, 2018). However, we believe that even low doses of Ayahuasca used in a chronically exposure regime were responsible for hampering the mnemonic processes in zebrafish. Nevertheless, further studies are still needed to evaluate potential harmful effects of prolonged exposure.

An alternative hypothesis can be drawn considering that after 13-days of Ayahuasca treatment, zebrafish were tested under abstinence of the drug. The withdrawal syndrome is typified mostly by an array of cognitive, behavioral and physiological symptoms (Favaro and de Paula, 2012). The increased locomotion reported for the chronically treated animals are compatible to this idea. This assertion occurs for we consider that the chronic groups lacked the drug on the test days, thus cognitive impairment may have derived from the withdrawal and not from excessive stimulation by DMT and β-cabolines. However, the absence of freezing (a typical anxiety related behavior; (Kalueff et al., 2013) did not accompany the symptoms (Figure 4), indicating that the withdrawal syndrome diagnostic cannot be ensured. Hence, the withdrawal or intoxication interpretation would need further investigation, preferably involving physiological parameters evaluation (i.e., cortisol), to be accepted or completely denied. Furthermore, Ayahuasca does not act in the reward system itself, and thus abstinence is not our main hypothesis in terms of the drug’s mechanism of action.

Finally, the Ayahuasca exposure regimes and concentrations used herein indicates significant modulation on the locomotor parameters and environmental exploration and discrimination, suggesting impaired cognitive performance of the chronic exposure while low acute concentrations seem to not affect one-trial discrimination in zebrafish. However, our knowledge on Ayahuasca’s potential to affect cognitive processes is still very little, and other exposure regimes and periods of withdrawal following the drug usage should be evaluated. For example, Ayahuasca long-term consumers use the drug once or twice a month, thus in a regime that cannot be properly comparable to the one used in the present study. In addition, cognitive protocols focusing in longer periods of learning would be important to better comprehend the extent of the mnemonic hampering observed in the one-trial learning in the present study. For future studies, we also suggest the use of zebrafish to test Ayahuasca exposure regimes on the development of drug seeking behavior. Studies have shown that regular ingestion of the brew is associated with a reduction in the consumption of abusive drugs (Fábregas et al., 2010; Thomas et al., 2013; Oliveira-lima et al., 2015; Soler et al., 2016), and this effect should be related to Ayahuasca action. Also, it would be important to invest in techniques that show changes in the brain (neurotransmitters, proteins, neuroplasticity) caused by Ayahuasca, in order to thoroughly understand the comparative effect of low vs. high and acute vs. chronic exposure of a drug that has been increasing used worldwide.

Overall, we reiterate the valuable contribution of the zebrafish in biomedical research of psychoactive drugs and showed that acute use of low Ayahuasca concentrations does not interfere on the memory formation of a single event, but prolonged exposure to concentrations considered as low may cause harmful effects to learning and memory processes. As such, Ayahuasca regime of exposure should be carefully considered, specially its use on a daily or weekly basis.

## Author Contributions

BL-S, AL and PS: conceived and designed the experiments. PE-S and HA: performed the experiments. JP-S and ACL: analyzed the data. BL-S: contributed reagents, materials, and analysis tools. BL-S, JP-S, PS and AL: wrote the article.

## Conflict of Interest Statement

The authors declare that the research was conducted in the absence of any commercial or financial relationships that could be construed as a potential conflict of interest.

## References

[B1] Akkerman S. Blokland A. Reneerkens O. van Goethem N. P. Bollen E. Gijselaers H. J. M.. (2012). Object recognition testing: methodological considerations on exploration and discrimination measures. Behav. Brain Res. 232, 335–347. 10.1016/j.bbr.2012.03.022 22490364

[B2] Alhaider A. A. Ageel A. M. Ginawi O. T. (1993). The quipazine-and YFMPP-increased conditioned avoidance response in rats: role of 5-HT_1C_/5-HT_2_ receptors. Neuropharmacology 32, 1427–1432. 10.1016/0028-3908(93)90040-a 8152532

[B3] Atucha E. Vukojevic V. Fornari R. V. Ronzoni G. Demougin P. Peter F.. (2017). Noradrenergic activation of the basolateral amygdala maintains hippocampus-dependent accuracy of remote memory. Proc. Natl. Acad. Sci. U S A 114, 9176–9181. 10.1073/pnas.1710819114 28790188 PMC5576838

[B4] Avdesh A. Martin-Iverson M. T. Mondal A. Chen M. Verdile G. Martins R. N. (2010). Natural colour preference in the zebrafish (*Danio rerio*). Proc. Meas. Behav. 2010, 155–157.

[B5] Barker G. R. I. Warburton E. C. (2011). When is the hippocampus involved in recognition memory? J. Neurosci. 31, 10721–10731. 10.1523/jneurosci.6413-10.2011 21775615 PMC6622630

[B6] Beitz A. J. Saxon D. (2004). Harmaline-induced climbing fiber activation causes amino acid and peptide release in the rodent cerebellar cortex and a unique temporal pattern of Fos expression in the olivo-cerebellar pathway. J. Neurocytol. 33, 49–74. 10.1023/b:neur.0000029648.81071.20 15173632

[B7] Bouso J. C. González D. Fondevila S. Cutchet M. Fernández X. Ribeiro Barbosa P. C.. (2012). Personality, psychopathology, life attitudes and neuropsychological performance among ritual users of ayahuasca: a longitudinal study. PLoS One 7:e42421. 10.1371/journal.pone.0042421 22905130 PMC3414465

[B8] Cai S. Huang S. Hao W. (2015). New hypothesis and treatment targets of depression: an integrated view of key findings. Neurosci. Bull. 31, 61–74. 10.1007/s12264-014-1486-4 25575479 PMC5562637

[B9] Carbonaro T. M. Gatch M. B. (2016). Neuropharmacology of N,N-dimethyltryptamine. Brain Res. Bull. 126, 74–88. 10.1016/j.brainresbull.2016.04.016 27126737 PMC5048497

[B10] Cassaday H. J. Hodges H. Gray J. A. (1993). The effects of ritanserin, RU 24969 and 8-OH-DPAT on latent inhibition in the rat. J. Psychopharmacol. 7, 63–71. 10.1177/026988119300700110 22290372

[B11] de Castro-Neto E. F. da Cunha R. H. da Silveira D. X. Yonamine M. Gouveia T. L. Cavalheiro E. A.. (2013). Changes in aminoacidergic and monoaminergic neurotransmission in the hippocampus and amygdala of rats after ayahuasca ingestion. World J. Biol. Chem. 4, 141–147. 10.4331/wjbc.v4.i4.141 24340137 PMC3856309

[B12] Chacon D. M. Luchiari A. C. (2014). A dose for the wiser is enough: the alcohol benefits for associative learning in zebrafish. Prog. Neuropsychopharmacol. Biol. Psychiatry 53, 109–115. 10.1016/j.pnpbp.2014.03.009 24681197

[B13] Davidson T. L. Hargrave S. L. Kearns D. N. Clasen M. M. Jones S. Wakeford A. G. P.. (2018). Cocaine impairs serial-feature negative learning and blood-brain barrier integrity. Pharmacol. Biochem. Behav. 170, 56–63. 10.1016/j.pbb.2018.05.005 29753886

[B14] dos Santos R. G. Hallak J. E. C. (2017). Effects of the natural β-carboline alkaloid harmine, a main constituent of ayahuasca, in memory and in the hippocampus: a systematic literature review of preclinical studies. J. Psychoactive Drugs 49, 1–10. 10.1080/02791072.2016.1260189 27918874

[B15] Fábregas J. M. González D. Fondevila S. Cutchet M. Fernández X. Barbosa P. C. R.. (2010). Assessment of addiction severity among ritual users of ayahuasca. Drug Alcohol Depend. 111, 257–261. 10.1016/j.drugalcdep.2010.03.024 20554400

[B16] Faraco J. H. Appelbaum L. Marin W. Gaus S. E. Mourrain P. Mignot E. (2006). Regulation of hypocretin (orexin) expression in embryonic zebrafish. J. Biol. Chem. 281, 29753–29761. 10.1074/jbc.M605811200 16867991

[B17] Favaro F. de Paula S. R. (2012). Dependentes químicos: o perfil da abstinência de drogas. J. Heal. Sci. Inst. 30, 41–43.

[B18] Figueroa A. R. M. (2012). Avaliação Dos Efeitos Neurotóxicos Do chá Ayahuasca Doctoral dissertation. São Paulo: Universidade de São Paulo.

[B19] França A. S. C. Lobão-Soares B. Muratori L. Nascimento G. Winne J. Pereira C. M.. (2015). D2 dopamine receptor regulation of learning, sleep and plasticity. Eur. Neuropsychopharmacol. 25, 493–504. 10.1016/j.euroneuro.2015.01.011 25778861

[B21] Frecska E. Bokor P. Winkelman M. (2016). The therapeutic potentials of ayahuasca: possible effects against various diseases of civilization. Front. Pharmachol. 7:35. 10.3389/fphar.2016.00035 26973523 PMC4773875

[B20] Frecska E. Móré C. E. Vargha A. Luna L. E. (2012). Enhancement of creative expression and entoptic phenomena as after-effects of repeated ayahuasca ceremonies. J. Psychoactive Drugs 44, 191–199. 10.1080/02791072.2012.703099 23061318

[B22] Fuller R. W. Warren B. J. Molloy B. B. (1970). Selective inhibition of monoamine oxidase in rat brain mitochondria. Biochem. Pharmacol. 19, 2934–2936. 10.1016/0006-2952(70)90036-5 5512701

[B23] Gerlai R. Lahav M. Guo S. Rosenthal A. (2000). Drinks like a fish: zebra fish *(Danio rerio)* as a behavior genetic model to study alcohol effects. Pharmacol. Biochem. Behav. 67, 773–782. 10.1016/s0091-3057(00)00422-6 11166068

[B24] Ghosh S. Reuveni I. Barkai E. Lamprecht R. (2016). Simultaneous and persistent CaMKII, PKC and PKA activation is required for maintaining learning-induced enhancement of AMPAR-mediated synaptic excitation. J. Neurochem. 136, 1107–1287. 10.1111/jnc.13505 26710089

[B25] Glennon R. A. Dukat M. Grella B. Hong S.-S. Costantino L. Teitler M.. (2000). Binding of β-carbolines and related agents at serotonin (5-HT_2_ and 5-HT_1A_), dopamine (D2) and benzodiazepine receptors. Drug Alcohol Depend. 60, 121–132. 10.1016/S0376-8716(99)00148-9 10940539

[B26] Good M. A. Barnes P. Staal V. McGregor A. Honey R. C. (2007). Context-but not familiarity-dependent forms of object recognition are impaired following excitotoxic hippocampal lesions in rats. Behav. Neurosci. 121, 218–223. 10.1037/0735-7044.121.1.218 17324066

[B27] Gould T. J. (2010). Addiction and cognition. Addict. Sci. Clin. Pract. 5, 4–14. 22002448 PMC3120118

[B28] Grella B. Teitler M. Smith C. Herrick-Davis K. Glennon R. A. (2003). Binding of β-carbolines at 5-HT_2_ serotonin receptors. Bioorg. Med. Chem. Lett. 13, 4421–4425. 10.1016/j.bmcl.2003.09.027 14643338

[B29] Grunwald D. J. Eisen J. S. (2002). Headwaters of the zebrafish—emergence of a new model vertebrate. Nat. Rev. Genet. 3, 717–724. 10.1038/nrg892 12209146

[B30] Harvey J. A. (2003). Role of the serotonin 5-HT_2A_ receptor in learning. Learn. Mem. 10, 355–362. 10.1101/lm.60803 14557608 PMC218001

[B31] Herculano A. M. Maximino C. (2014). Serotonergic modulation of zebrafish behavior: towards a paradox. Prog. Neuropsychopharmacol. Biol. Psychiatry 55, 50–66. 10.1016/j.pnpbp.2014.03.008 24681196

[B32] Holzschuh J. Ryu S. Aberger F. Driever W. (2001). Dopamine transporter expression distinguishes dopaminergic neurons from other catecholaminergic neurons in the developing zebrafish embryo. Mech. Dev. 101, 237–243. 10.1016/s0925-4773(01)00287-8 11231083

[B33] Jackson D. Bruno J. P. Stachowiak M. K. Michael J. Z. (1988). Inhibition of striatal acetylcholine release by serotonin and dopamine after the intracerebral administration of 6-hydroxydopamine to neonatal rats. Brain Res. 457, 267–273. 10.1016/0006-8993(88)90695-6 3146404

[B34] Jäkälä P. Sirviö J. Jolkkonen J. Riekkinen P. Jr. Acsady L. Riekkinen P. (1992). The effects of p-chlorophenylalanine-induced serotinin synthesis inhibition and muscarinic blockade on the performance of rats in a 5-choice serial reaction time task. Behav. Brain Res. 51, 29–40. 10.1016/s0166-4328(05)80309-2 1282817

[B35] Jiang X. L. Shen H. W. Yu A. M. (2015). Potentiation of 5-methoxy-N,N-dimethyltryptamine-induced hyperthermia by harmaline and the involvement of activation of 5-HT_1A_ and 5-HT_2A_ receptors. Neuropharmacology 89, 342–351. 10.1016/j.neuropharm.2014.10.013 25446678 PMC4310233

[B36] Johnstad P. G. (2018). Powerful substances in tiny amounts: an interview study of psychedelic microdosing. Nord. Stud. Alcohol Drugs 35, 39–51. 10.1177/1455072517753339 PMC7434115 32934512

[B37] Kalueff A. V. Gebhardt M. Stewart A. M. Cachat J. M. Brimmer M. Chawla J. S.. (2013). Towards a comprehensive catalog of zebrafish behavior 1.0 and beyond. Zebrafish 10, 70–86. 10.1089/zeb.2012.0861 23590400 PMC3629777

[B38] Kalueff A. V. Stewart A. M. Gerlai R. (2014). Zebrafish as an emerging model for studying complex brain disorders. Trends Pharmacol. Sci. 35, 63–75. 10.1016/j.tips.2013.12.002 24412421 PMC3913794

[B39] Kaslin J. Panula P. (2001). Comparative anatomy of the histaminergic and other aminergic systems in zebrafish (*Danio rerio*). J. Comp. Neurol. 440, 342–377. 10.1002/cne.1390 11745628

[B40] Kaslin J. Nystedt J. M. Ostergård M. Peitsaro N. Panula P. (2004). The orexin/hypocretin system in zebrafish is connected to the aminergic and cholinergic systems. J. Neurosci. 24, 2678–2689. 10.1523/JNEUROSCI.4908-03.2004 15028760 PMC6729510

[B41] Kolb B. Whishaw I. Q. (1998). Brain plasticity and behavior. Annu. Rev. Psychol. 49, 43–64. 10.1146/annurev.psych.49.1.43 9496621

[B42] Kralic J. E. Criswell H. E. Osterman J. L. O’Buckley T. K. Wilkie M. E. Matthews D. B.. (2005). Genetic essential tremor in γ-aminobutyric acid A receptor α1 subunit knockout mice. J. Clin. Invest. 115, 774–779. 10.1172/jci23625 15765150 PMC1052003

[B43] Kruschke J. K. Aguinis H. Joo H. (2012). The time has come: bayesian methods for data analysis in the organizational sciences. Organ. Res. Methods 15, 722–752. 10.1177/1094428112457829

[B44] Kuypers K. P. C. Ramaekers J. G. (2007). Acute dose of MDMA (75 mg) impairs spatial memory for location but leaves contextual processing of visuospatial information unaffected. Psychopharmacology 189, 557–563. 10.1007/s00213-006-0321-7 16508761

[B45] Luchiari A. C. Chacon D. M. M. (2013). Physical exercise improves learning in zebrafish, *Danio rerio*. Behav. Processes 100, 44–47. 10.1016/j.beproc.2013.07.020 23933376

[B46] Luchiari A. C. Salajan D. C. Gerlai R. (2015). Acute and chronic alcohol administration: effects on performance of zebrafish in a latent learning task. Behav. Brain Res. 282, 76–83. 10.1016/j.bbr.2014.12.013 25557800 PMC4339105

[B47] Lucon-Xiccato T. Dadda M. (2014). Assessing memory in zebrafish using the one-trial test. Behav. Processes 106, 1–4. 10.1016/j.beproc.2014.03.010 24704579

[B48] May Z. Morrill A. Holcombe A. Johnston T. Gallup J. Fouad K.. (2016). Object recognition memory in zebrafish. Behav. Brain Res. 296, 199–210. 10.1016/j.bbr.2015.09.016 26376244

[B49] McKenna D. J. (2004). Clinical investigations of the therapeutic potential of ayahuasca: rationale and regulatory challenges. Pharmacol. Ther. 102, 111–129. 10.1016/j.pharmthera.2004.03.002 15163593

[B50] McKenna D. J. Towers G. H. N. Abbott F. (1984). Monoamine oxidase inhibitors in South American hallucinogenic plants: tryptamine and β-carboline constituents of Ayahuasca. J. Ethnopharmacol. 10, 195–223. 10.1016/0378-8741(84)90003-5 6587171

[B51] McLean D. L. Fetcho J. R. (2004). Relationship of tyrosine hydroxylase and serotonin immunoreactivity to sensorimotor circuitry in larval zebrafish. J. Comp. Neurol. 480, 57–71. 10.1002/cne.20281 15514919

[B52] Meneses A. (1999). 5-HT system and cognition. Neurosci. Biobehav. Rev. 23, 1111–1125. 10.1016/s0149-7634(99)00067-6 10643820

[B53] Miwa H. Nishi K. Fuwa T. Mizuno Y. (2000). Differential expression of c-fos following administration of two tremorgenic agents: harmaline and oxotremorine. Neuroreport 11, 2385–2390. 10.1097/00001756-200008030-00010 10943690

[B54] Mumby D. G. Glenn M. J. Nesbitt C. Kyriazis D. A. (2002). Dissociation in retrograde memory for object discriminations and object recognition in rats with perirhinal cortex damage. Behav. Brain Res. 132, 215–226. 10.1016/s0166-4328(01)00444-2 11997151

[B55] Oliveira J. Silveira M. Chacon D. Luchiari A. (2015). The zebrafish world of colors and shapes: preference and discrimination. Zebrafish 12, 166–173. 10.1089/zeb.2014.1019 25674976

[B57] Oliveira-lima A. J. Santos R. Hollais A. W. Gerardi-junior C. A. Baldaia M. A. Wuo-silva R.. (2015). Physiology and behavior effects of ayahuasca on the development of ethanol-induced behavioral sensitization and on a post-sensitization treatment in mice. Physiol. Behav. 142, 28–36. 10.1016/j.physbeh.2015.01.032 25637859

[B58] Paterson N. E. Malekiani S. A. Foreman M. M. Olivier B. Hanania T. (2009). Pharmacological characterization of harmaline-induced tremor activity in mice. Eur. J. Pharmacol. 616, 73–80. 10.1016/j.ejphar.2009.05.031 19497322

[B59] Pic-Taylor A. da Motta L. G. de Morais J. A. Junior W. M. Santos Ade F. A. Campos L. A.. (2015). Behavioural and neurotoxic effects of ayahuasca infusion (*Banisteriopsis caapi and Psychotria viridis*) in female Wistar rat. Behav. Processes 118, 102–110. 10.1016/j.beproc.2015.05.004 26049017

[B60] Pinheiro-da-Silva J. Silva P. F. Nogueira M. B. Luchiari A. C. (2017). Sleep deprivation effects on object discrimination task in zebrafish (*Danio rerio*). Anim. Cogn. 20, 159–169. 10.1007/s10071-016-1034-x 27646310

[B61] Potvin S. Pelletier J. Grot S. Hébert C. Barr A. Lecomte T. (2018). Cognitive deficits in individuals with methamphetamine use disorder: a meta-analysis. Addict. Behav. 80, 154–160. 10.1016/j.addbeh.2018.01.021 29407687

[B62] Prober D. A. Rihel J. Onah A. A. Sung R.-J. Schier A. F. (2006). Hypocretin/orexin overexpression induces an insomnia-like phenotype in zebrafish. J. Neurosci. 26, 13400–13410. 10.1523/JNEUROSCI.4332-06.2006 17182791 PMC6675014

[B63] Rabin R. A. Regina M. Doat M. Winter J. C. (2002). 5-HT_2A_ receptor-stimulated phosphoinositide hydrolysis in the stimulus effects of hallucinogens. Pharmacol. Biochem. Behav. 72, 29–37. 10.1016/s0091-3057(01)00720-1 11900766

[B64] Ray T. S. (2010). Psychedelics and the human receptorome. PLoS One 5:e9019. 10.1371/journal.pone.0009019 20126400 PMC2814854

[B65] Rico E. P. Rosemberg D. B. Dias R. D. Bogo M. R. Bonan C. D. (2007). Ethanol alters acetylcholinesterase activity and gene expression in zebrafish brain. Toxicol. Lett. 174, 25–30. 10.1016/j.toxlet.2007.08.005 17888594

[B66] Rodríguez F. López J. C. Vargas J. P. Gómez Y. Broglio C. Salas C. (2002). Conservation of spatial memory function in the pallial forebrain of reptiles and ray-finned fishes. J. Neurosci. 22, 2894–2903. 10.1523/JNEUROSCI.22-07-02894.2002 11923454 PMC6758289

[B67] Saar D. Grossman Y. Barkai E. (2002). Learning-induced enhancement of postsynaptic potentials in pyramidal neurons. J. Neurophysiol. 87, 2358–2363. 10.1152/jn.2002.87.5.2358 11976373

[B68] Santos L. C. Ruiz-Oliveira J. Oliveira J. J. Silva P. F. Luchiari A. C. (2016). Irish coffee: effects of alcohol and caffeine on object discrimination in zebrafish. Pharmacol. Biochem. Behav. 143, 34–43. 10.1016/j.pbb.2016.01.013 26850919

[B69] Savoldi R. Polari D. Pinheiro-da-Silva J. Silva P. F. Lobao-Soares B. Yonamine M.. (2017). Behavioral changes over time following ayahuasca exposure in zebrafish. Front. Behav. Neurosci. 11:139. 10.3389/fnbeh.2017.00139 28804451 PMC5532431

[B70] Sison M. Gerlai R. (2010). Associative learning in zebrafish (*Danio rerio*) in the plus maze. Behav. Brain Res. 207, 99–104. 10.1016/j.bbr.2009.09.043 19800919 PMC2814798

[B71] Soler J. Elices M. Franquesa A. Barker S. (2016). Exploring the therapeutic potential of Ayahuasca: acute intake increases mindfulness-related capacities. Psychopharmacology 233, 823–829. 10.1007/s00213-015-4162-0 26612618

[B72] Sparks D. W. Tian M. K. Sargin D. Venkatesan S. Intson K. Lambe E. K. (2018). Opposing cholinergic and serotonergic modulation of layer 6 in prefrontal cortex. Front. Neural Circuits 11:107. 10.3389/fncir.2017.00107 29354034 PMC5758509

[B73] Sterling M. E. Karatayev O. Chang G. Q. Algava D. B. Leibowitz S. F. (2015). Model of voluntary ethanol intake in zebrafish: effect on behavior and hypothalamic orexigenic peptides. Behav. Brain Res. 278, 29–39. 10.1016/j.bbr.2014.09.024 25257106 PMC4369451

[B74] Stewart A. M. Ullmann J. F. P. Norton W. H. J. Parker M. O. Brennan C. H. Gerlai R.. (2014). Molecular psychiatry of zebrafish. Mol. Psychiatry 20, 2–17. 10.1038/mp.2014.128 25349164 PMC4318706

[B75] Thomas G. Lucas P. Capler N. R. Tupper K. W. Martin G. (2013). Ayahuasca-assisted therapy for addiction: results from a preliminary observational study in Canada. Curr. Drug Abuse Rev. 6, 30–42. 10.2174/15733998113099990003 23627784

[B76] Vik P. W. Cellucci T. Jarchow A. Hedt J. (2004). Cognitive impairment in substance abuse. Psychiatr. Clin. North Am. 27, 97–109. 10.1016/S0193-953X(03)00110-2 15062633

[B77] Vishnoi S. Raisuddin S. Parvez S. (2016). Behavioral tagging: a novel model for studying long-term memory. Neurosci. Biobehav. Rev. 68, 361–369. 10.1016/j.neubiorev.2016.05.017 27216211

[B78] White N. M. Packard M. G. McDonald R. J. (2013). Dissociation of memory systems: the story unfolds. Behav. Neurosci. 127, 813–834. 10.1037/a0034859 24341707

[B79] Yasuhara H. Sho S. Kamijo K. (1972). Difference in actions of harmine on the oxidations of serotonin and tyramine by beef brain mitochondrial MAO. Jpn. J. Pharmacol. 22, 439–441. 10.1254/jjp.22.439 4539405

[B80] Zuur A. F. Ieno E. N. Elphick C. S. (2010). A protocol for data exploration to avoid common statistical problems. Methods Ecol. Evol. 1, 3–14. 10.1111/j.2041-210x.2009.00001.x

