# Supplementary material for: It’s Tea Time: Interference of Ayahuasca Brew on Discriminative Learning in Zebrafish
Source: Front Behav Neurosci. 2018 Aug 27;12:190. doi: 10.3389/fnbeh.2018.00190 (PMC6119691; doi:10.3389/fnbeh.2018.00190)
Supplement: Supplementary file 1 [file Image_1.pdf]

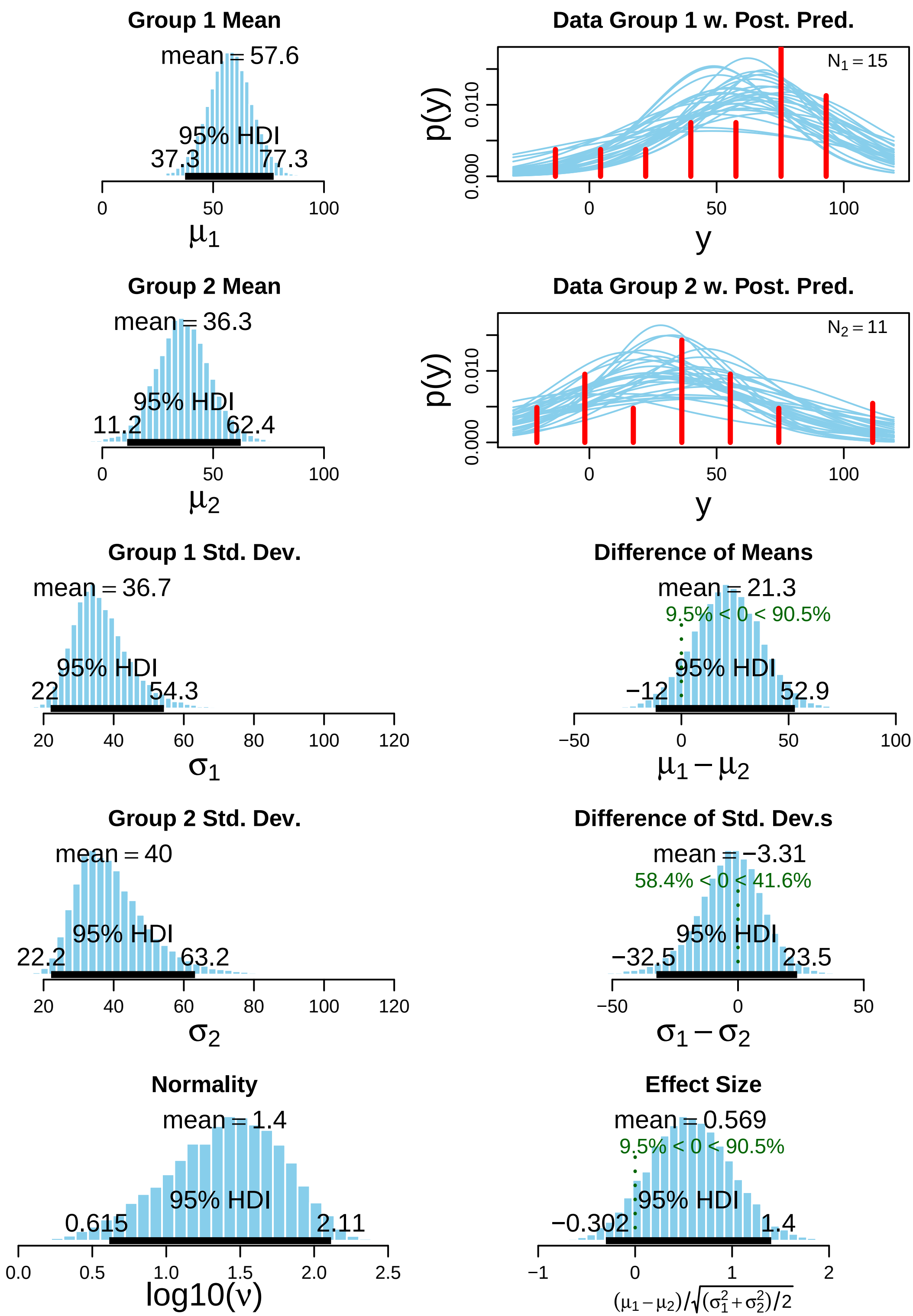
Group 1 Mean
mean = 57.6
95% HDI
37.3
77.3
0
50
100
μ1
Data Group 1 w. Post. Pred.
N1 = 15
p(y)
0.000
0.010
0
50
100
y
Group 2 Mean
mean = 36.3
95% HDI
11.2
62.4
0
50
100
μ2
Data Group 2 w. Post. Pred.
N2 = 11
p(y)
0.000
0.010
0
50
100
y
Group 1 Std. Dev.
mean = 36.7
95% HDI
22
54.3
20
40
60
80
100
120
σ1
Difference of Means
mean = 21.3
9.5% < 0 < 90.5%
95% HDI
−12
52.9
−50
0
50
100
μ1 − μ2
Group 2 Std. Dev.
mean = 40
95% HDI
22.2
63.2
20
40
60
80
100
120
σ2
Difference of Std. Dev.s
mean = −3.31
58.4% < 0 < 41.6%
95% HDI
−32.5
23.5
−50
0
50
σ1 − σ2
Normality
mean = 1.4
95% HDI
0.615
2.11
0.0
0.5
1.0
1.5
2.0
2.5
log10(ν)
Effect Size
mean = 0.569
9.5% < 0 < 90.5%
95% HDI
−0.302
1.4
−1
0
1
2
(μ1 − μ2)/√((σ1² + σ2²)/2)

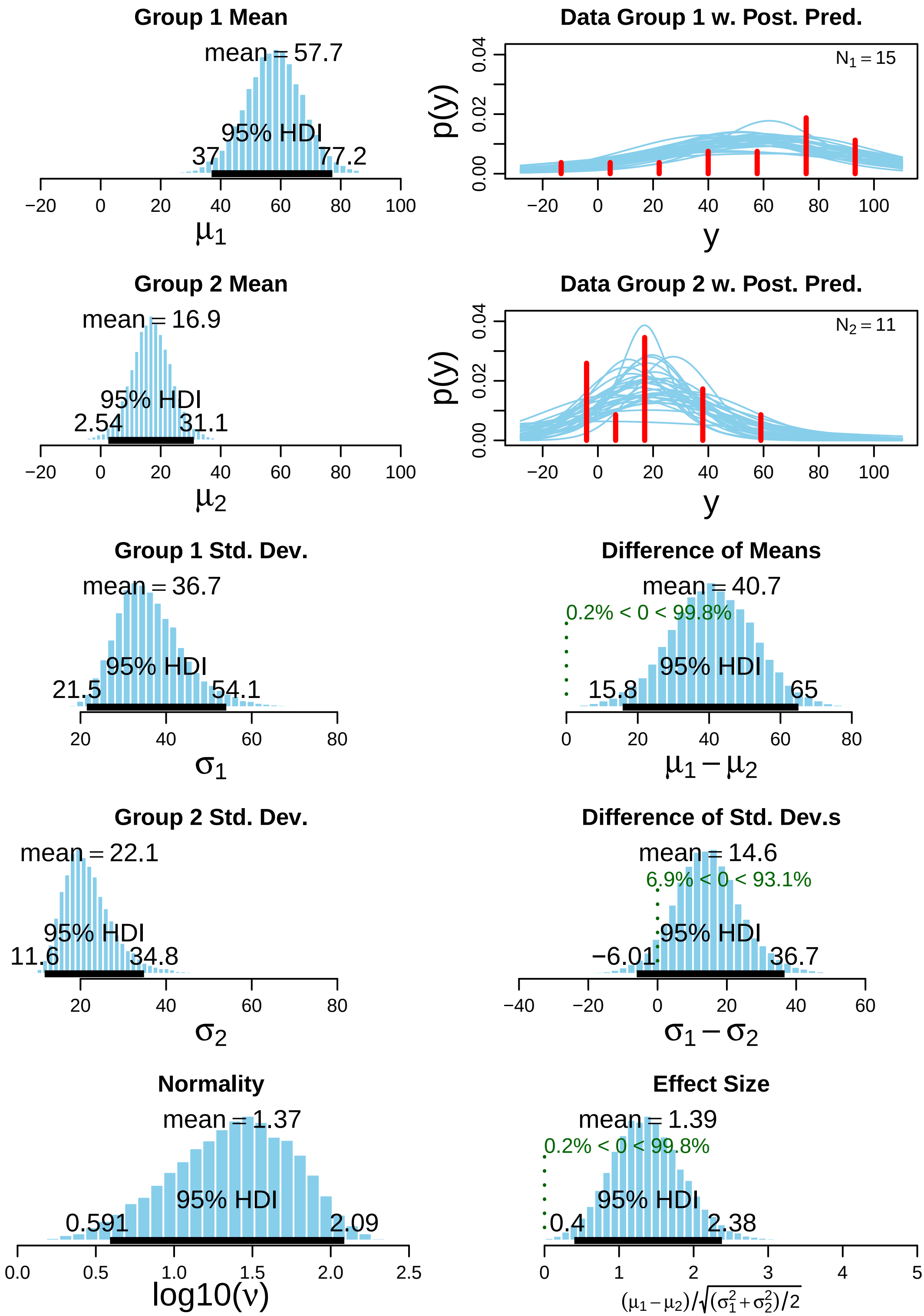
Group 1 Mean
mean = 57.7
95% HDI
37
77.2
−20 0 20 40 60 80 100
μ1
Data Group 1 w. Post. Pred.
N1 = 15
p(y)
0.00 0.02 0.04
−20 0 20 40 60 80 100
y
Group 2 Mean
mean = 16.9
95% HDI
2.54
31.1
−20 0 20 40 60 80 100
μ2
Data Group 2 w. Post. Pred.
N2 = 11
p(y)
0.00 0.02 0.04
−20 0 20 40 60 80 100
y
Group 1 Std. Dev.
mean = 36.7
95% HDI
21.5
54.1
20 40 60 80
σ1
Difference of Means
mean = 40.7
0.2% < 0 < 99.8%
95% HDI
15.8
65
0 20 40 60 80
μ1 − μ2
Group 2 Std. Dev.
mean = 22.1
95% HDI
11.6
34.8
20 40 60 80
σ2
Difference of Std. Dev.s
mean = 14.6
6.9% < 0 < 93.1%
95% HDI
−6.01
36.7
−40 −20 0 20 40 60
σ1 − σ2
Normality
mean = 1.37
95% HDI
0.591
2.09
0.0 0.5 1.0 1.5 2.0 2.5
log10(ν)
Effect Size
mean = 1.39
0.2% < 0 < 99.8%
95% HDI
0.4
2.38
0 1 2 3 4 5
(μ1 − μ2)/√((σ1² + σ2²)/2)

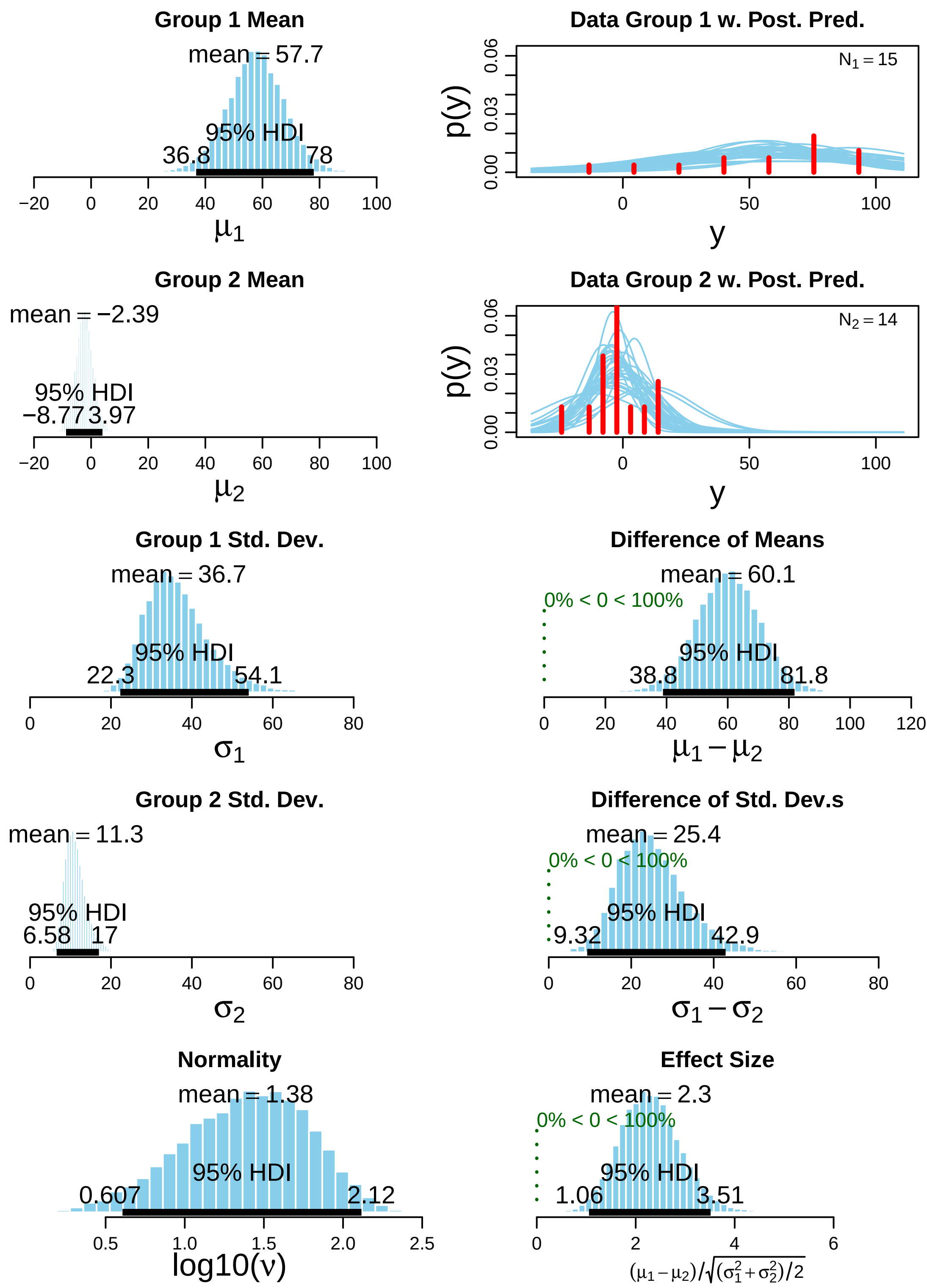

Group 1 Mean
mean = 57.7
95% HDI
36.8
78
−20 0 20 40 60 80 100
μ1
Data Group 1 w. Post. Pred.
N1 = 15
p(y)
0.00 0.03 0.06
0 50 100
y
Group 2 Mean
mean = −2.39
95% HDI
−8.77
3.97
−20 0 20 40 60 80 100
μ2
Data Group 2 w. Post. Pred.
N2 = 14
p(y)
0.00 0.03 0.06
0 50 100
y
Group 1 Std. Dev.
mean = 36.7
95% HDI
22.3
54.1
0 20 40 60 80
σ1
Difference of Means
mean = 60.1
0% < 0 < 100%
95% HDI
38.8
81.8
0 20 40 60 80 100 120
μ1 − μ2
Group 2 Std. Dev.
mean = 11.3
95% HDI
6.58
17
0 20 40 60 80
σ2
Difference of Std. Dev.s
mean = 25.4
0% < 0 < 100%
95% HDI
9.32
42.9
0 20 40 60 80
σ1 − σ2
Normality
mean = 1.38
95% HDI
0.607
2.12
0.5 1.0 1.5 2.0 2.5
log10(ν)
Effect Size
mean = 2.3
0% < 0 < 100%
95% HDI
1.06
3.51
0 2 4 6
(μ1 − μ2)/√((σ1² + σ2²)/2)

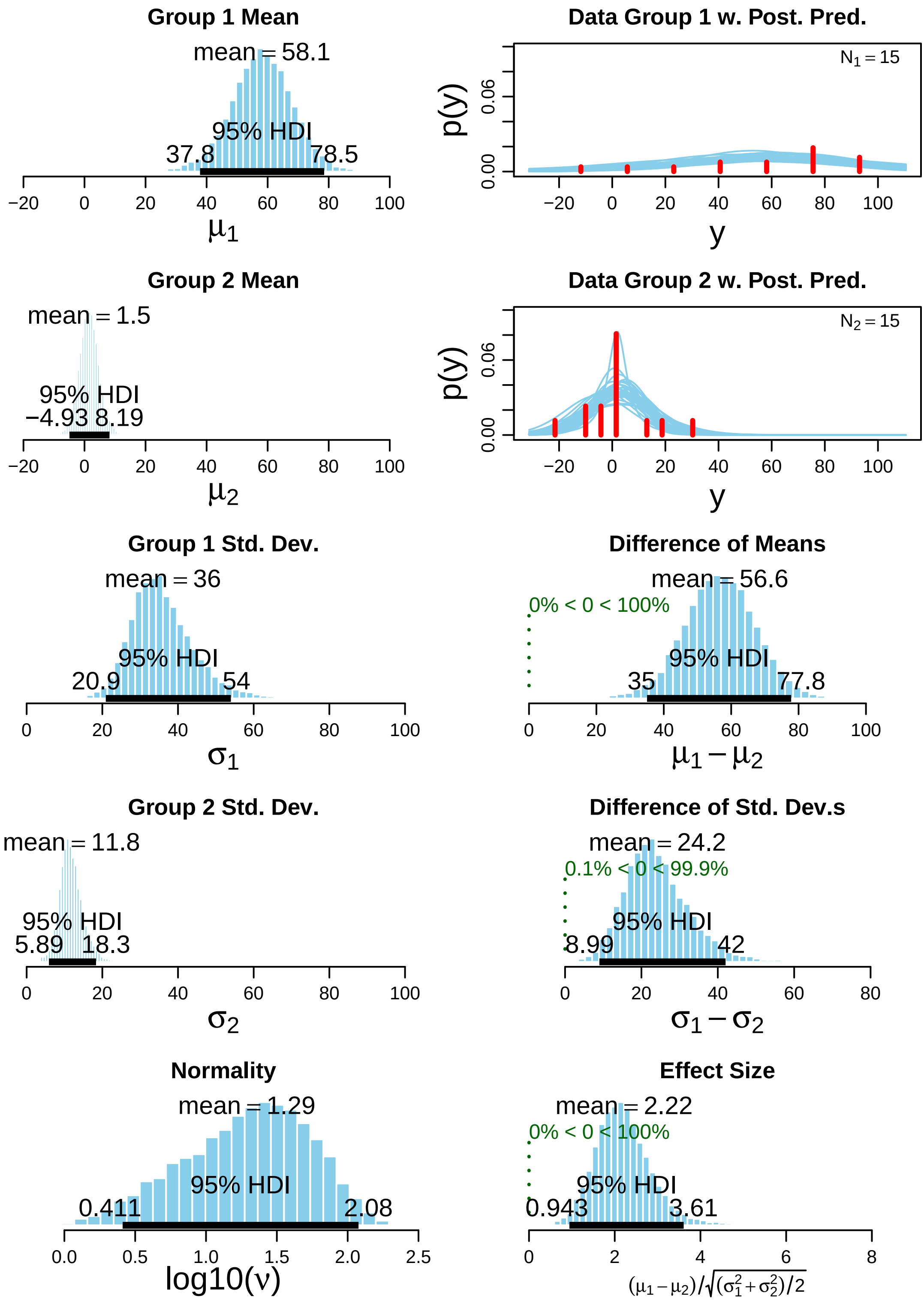
Group 1 Mean
mean = 58.1
95% HDI
37.8
78.5
−20 0 20 40 60 80 100
μ1
Data Group 1 w. Post. Pred.
N1 = 15
p(y)
0.00 0.06
−20 0 20 40 60 80 100
y
Group 2 Mean
mean = 1.5
95% HDI
−4.93 8.19
−20 0 20 40 60 80 100
μ2
Data Group 2 w. Post. Pred.
N2 = 15
p(y)
0.00 0.06
−20 0 20 40 60 80 100
y
Group 1 Std. Dev.
mean = 36
95% HDI
20.9
54
0 20 40 60 80 100
σ1
Difference of Means
mean = 56.6
0% < 0 < 100%
95% HDI
35
77.8
0 20 40 60 80 100
μ1 − μ2
Group 2 Std. Dev.
mean = 11.8
95% HDI
5.89
18.3
0 20 40 60 80 100
σ2
Difference of Std. Dev.s
mean = 24.2
0.1% < 0 < 99.9%
95% HDI
8.99
42
0 20 40 60 80
σ1 − σ2
Normality
mean = 1.29
95% HDI
0.411
2.08
0.0 0.5 1.0 1.5 2.0 2.5
log10(ν)
Effect Size
mean = 2.22
0% < 0 < 100%
95% HDI
0.943
3.61
0 2 4 6 8
(μ1 − μ2)/√((σ1² + σ2²)/2)

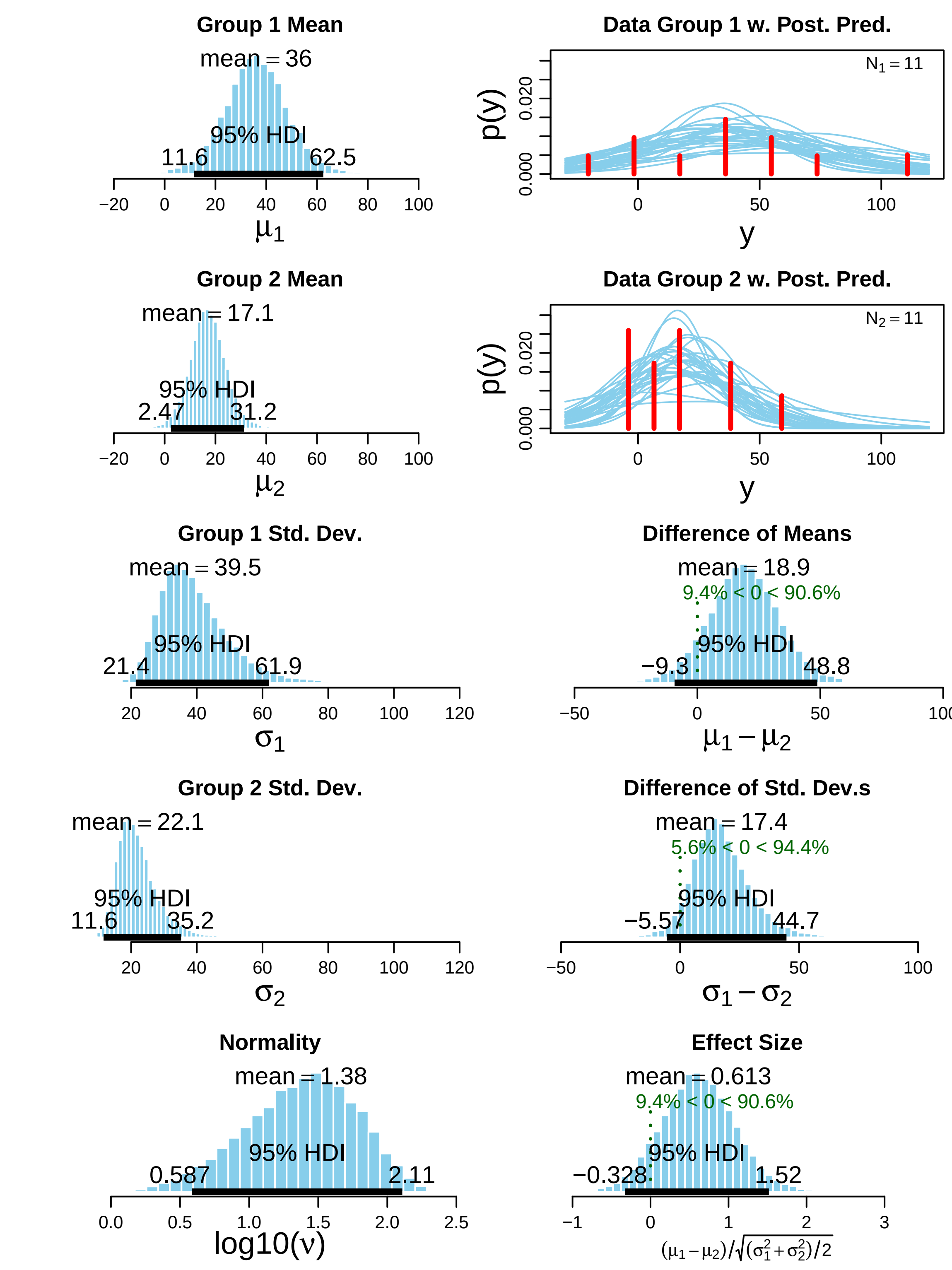

Group 1 Mean
mean = 36
95% HDI
11.6
62.5
μ_1
Data Group 1 w. Post. Pred.
$N_1 = 11$
p(y)
y
Group 2 Mean
mean = 17.1
95% HDI
2.47
31.2
μ_2
Data Group 2 w. Post. Pred.
$N_2 = 11$
p(y)
y
Group 1 Std. Dev.
mean = 39.5
95% HDI
21.4
61.9
σ_1
Difference of Means
mean = 18.9
9.4% < 0 < 90.6%
95% HDI
−9.3
48.8
$\mu_1 - \mu_2$
Group 2 Std. Dev.
mean = 22.1
95% HDI
11.6
35.2
σ_2
Difference of Std. Dev.s
mean = 17.4
5.6% < 0 < 94.4%
95% HDI
−5.57
44.7
$\sigma_1 - \sigma_2$
Normality
mean = 1.38
95% HDI
0.587
2.11
log10(ν)
Effect Size
mean = 0.613
9.4% < 0 < 90.6%
95% HDI
−0.328
1.52
$(\mu_1 - \mu_2)/\sqrt{(\sigma_1^2 + \sigma_2^2)/2}$

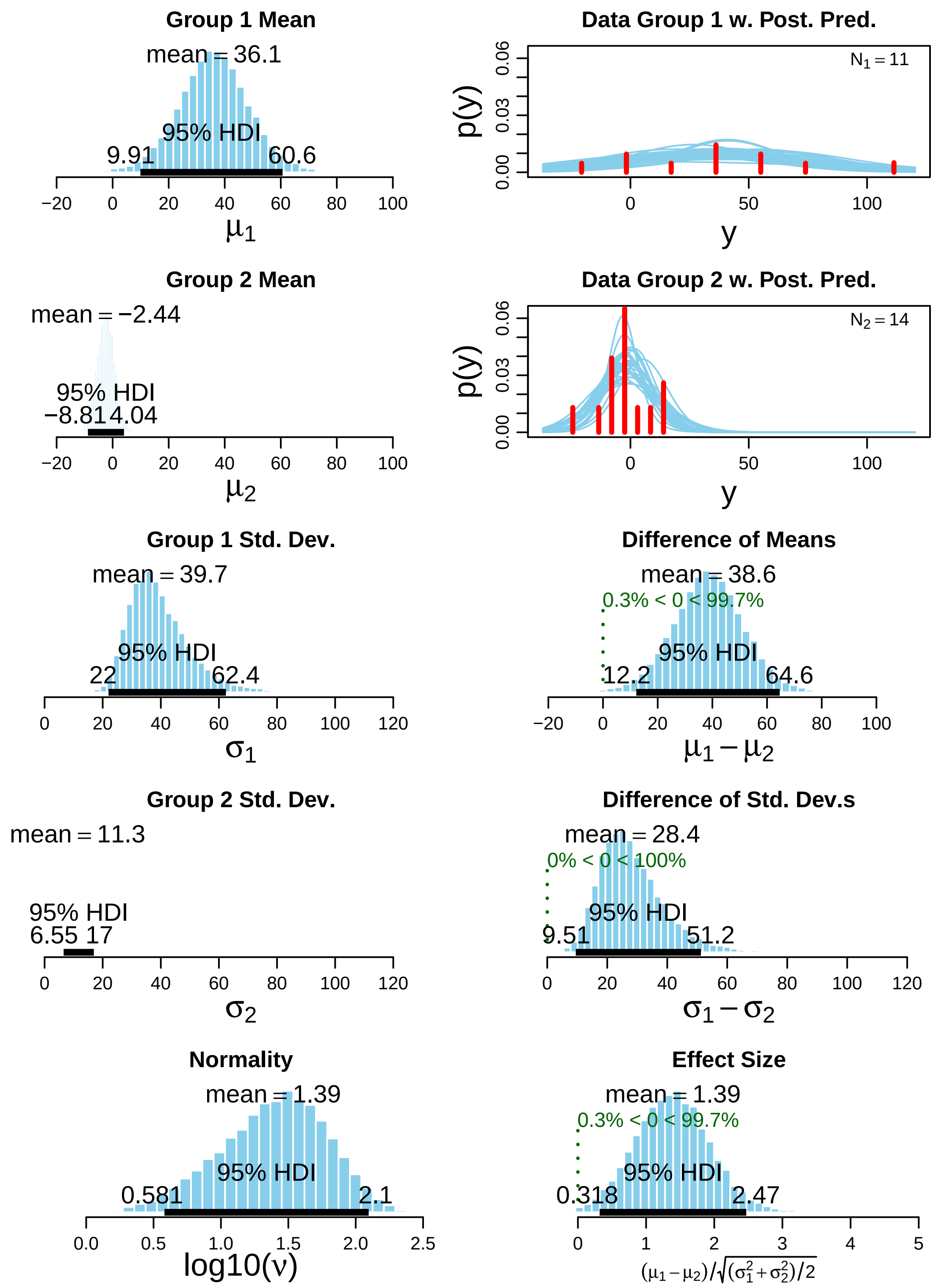
Group 1 Mean
mean = 36.1
95% HDI
9.91
60.6
−20 0 20 40 60 80 100
μ1
Data Group 1 w. Post. Pred.
N1 = 11
p(y)
0.00 0.03 0.06
0 50 100
y
Group 2 Mean
mean = −2.44
95% HDI
−8.81 4.04
−20 0 20 40 60 80 100
μ2
Data Group 2 w. Post. Pred.
N2 = 14
p(y)
0.00 0.03 0.06
0 50 100
y
Group 1 Std. Dev.
mean = 39.7
95% HDI
22
62.4
0 20 40 60 80 100 120
σ1
Difference of Means
mean = 38.6
0.3% < 0 < 99.7%
95% HDI
12.2
64.6
−20 0 20 40 60 80 100
μ1 − μ2
Group 2 Std. Dev.
mean = 11.3
95% HDI
6.55 17
0 20 40 60 80 100 120
σ2
Difference of Std. Dev.s
mean = 28.4
0% < 0 < 100%
95% HDI
9.51
51.2
0 20 40 60 80 100 120
σ1 − σ2
Normality
mean = 1.39
95% HDI
0.581
2.1
0.0 0.5 1.0 1.5 2.0 2.5
log10(ν)
Effect Size
mean = 1.39
0.3% < 0 < 99.7%
95% HDI
0.318
2.47
0 1 2 3 4 5
(μ1 − μ2)/√((σ1² + σ2²)/2)

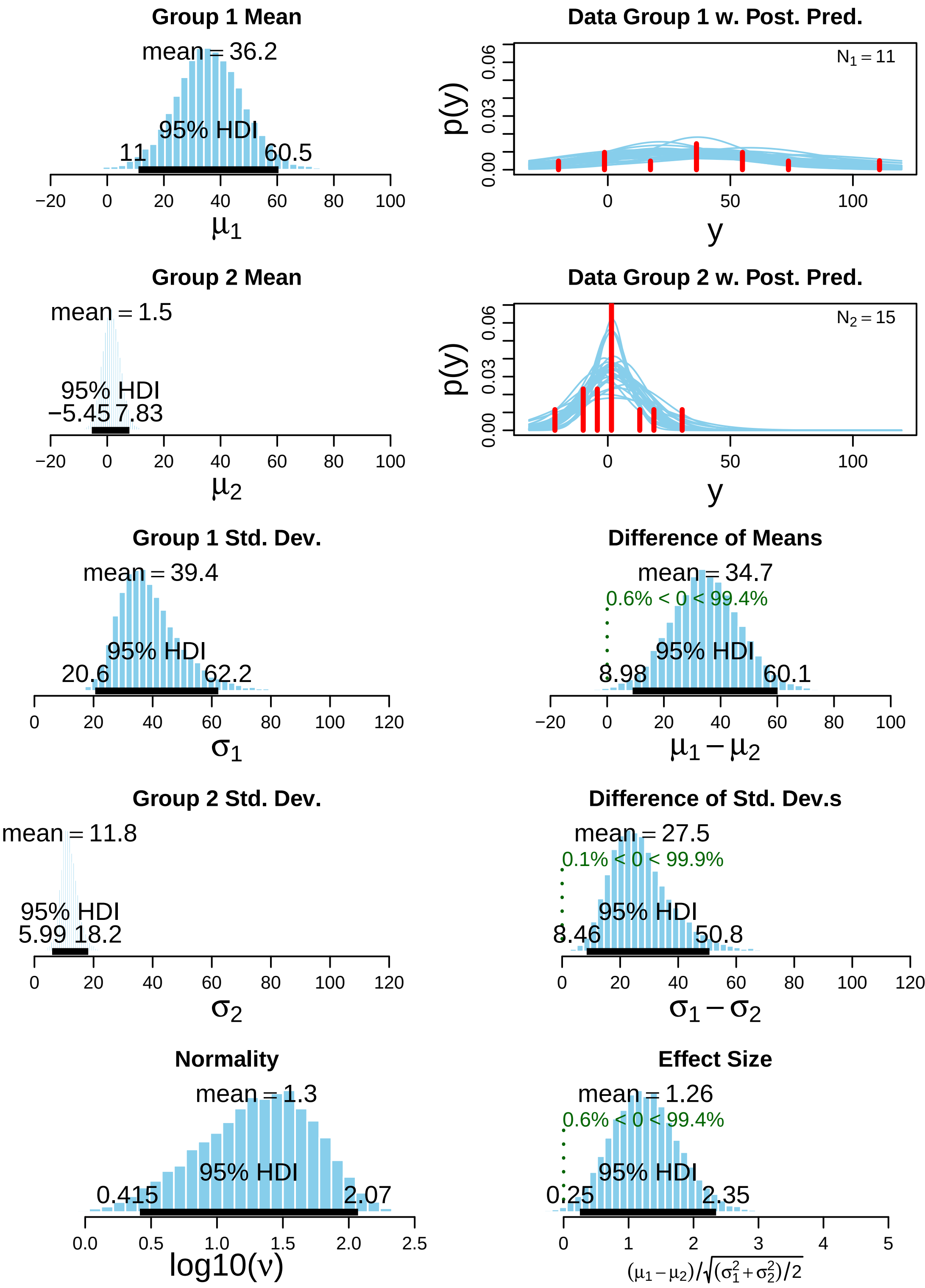

Group 1 Mean
mean = 36.2
95% HDI
11
60.5
−20 0 20 40 60 80 100
μ1
Data Group 1 w. Post. Pred.
N1 = 11
p(y)
0.00 0.03 0.06
0 50 100
y
Group 2 Mean
mean = 1.5
95% HDI
−5.45 7.83
−20 0 20 40 60 80 100
μ2
Data Group 2 w. Post. Pred.
N2 = 15
p(y)
0.00 0.03 0.06
0 50 100
y
Group 1 Std. Dev.
mean = 39.4
95% HDI
20.6
62.2
0 20 40 60 80 100 120
σ1
Difference of Means
mean = 34.7
0.6% < 0 < 99.4%
95% HDI
8.98
60.1
−20 0 20 40 60 80 100
μ1 − μ2
Group 2 Std. Dev.
mean = 11.8
95% HDI
5.99 18.2
0 20 40 60 80 100 120
σ2
Difference of Std. Dev.s
mean = 27.5
0.1% < 0 < 99.9%
95% HDI
8.46
50.8
0 20 40 60 80 100 120
σ1 − σ2
Normality
mean = 1.3
95% HDI
0.415
2.07
0.0 0.5 1.0 1.5 2.0 2.5
log10(ν)
Effect Size
mean = 1.26
0.6% < 0 < 99.4%
95% HDI
0.25
2.35
0 1 2 3 4 5
(μ1 − μ2)/√((σ1² + σ2²)/2)

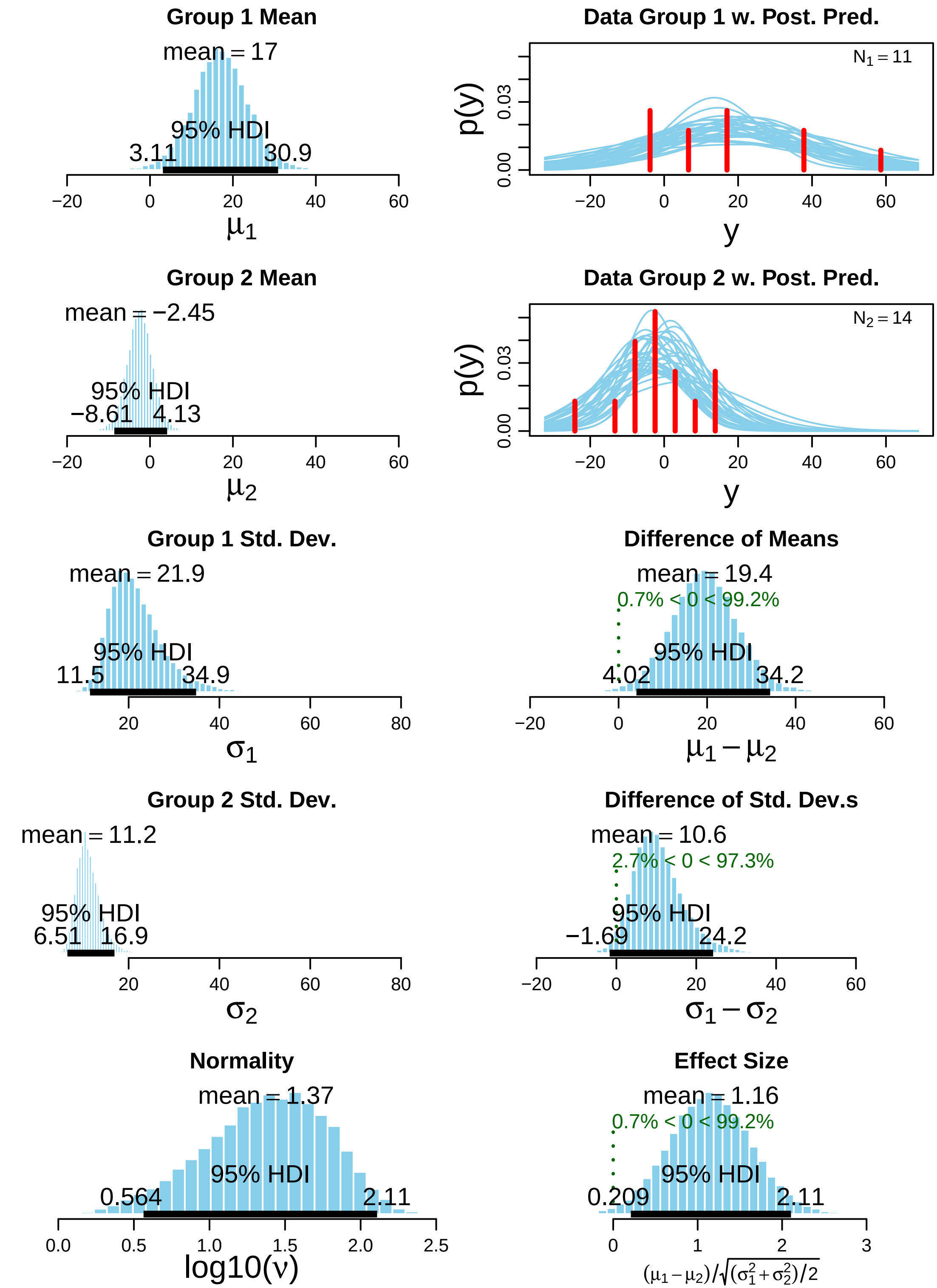

Group 1 Mean
mean = 17
95% HDI
3.11
30.9
μ1
Data Group 1 w. Post. Pred.
N1 = 11
p(y)
y
Group 2 Mean
mean = −2.45
95% HDI
−8.61
4.13
μ2
Data Group 2 w. Post. Pred.
N2 = 14
p(y)
y
Group 1 Std. Dev.
mean = 21.9
95% HDI
11.5
34.9
σ1
Difference of Means
mean = 19.4
0.7% < 0 < 99.2%
95% HDI
4.02
34.2
μ1 − μ2
Group 2 Std. Dev.
mean = 11.2
95% HDI
6.51
16.9
σ2
Difference of Std. Dev.s
mean = 10.6
2.7% < 0 < 97.3%
95% HDI
−1.69
24.2
σ1 − σ2
Normality
mean = 1.37
95% HDI
0.564
2.11
log10(ν)
Effect Size
mean = 1.16
0.7% < 0 < 99.2%
95% HDI
0.209
2.11
(μ1 − μ2)/√((σ1² + σ2²)/2)

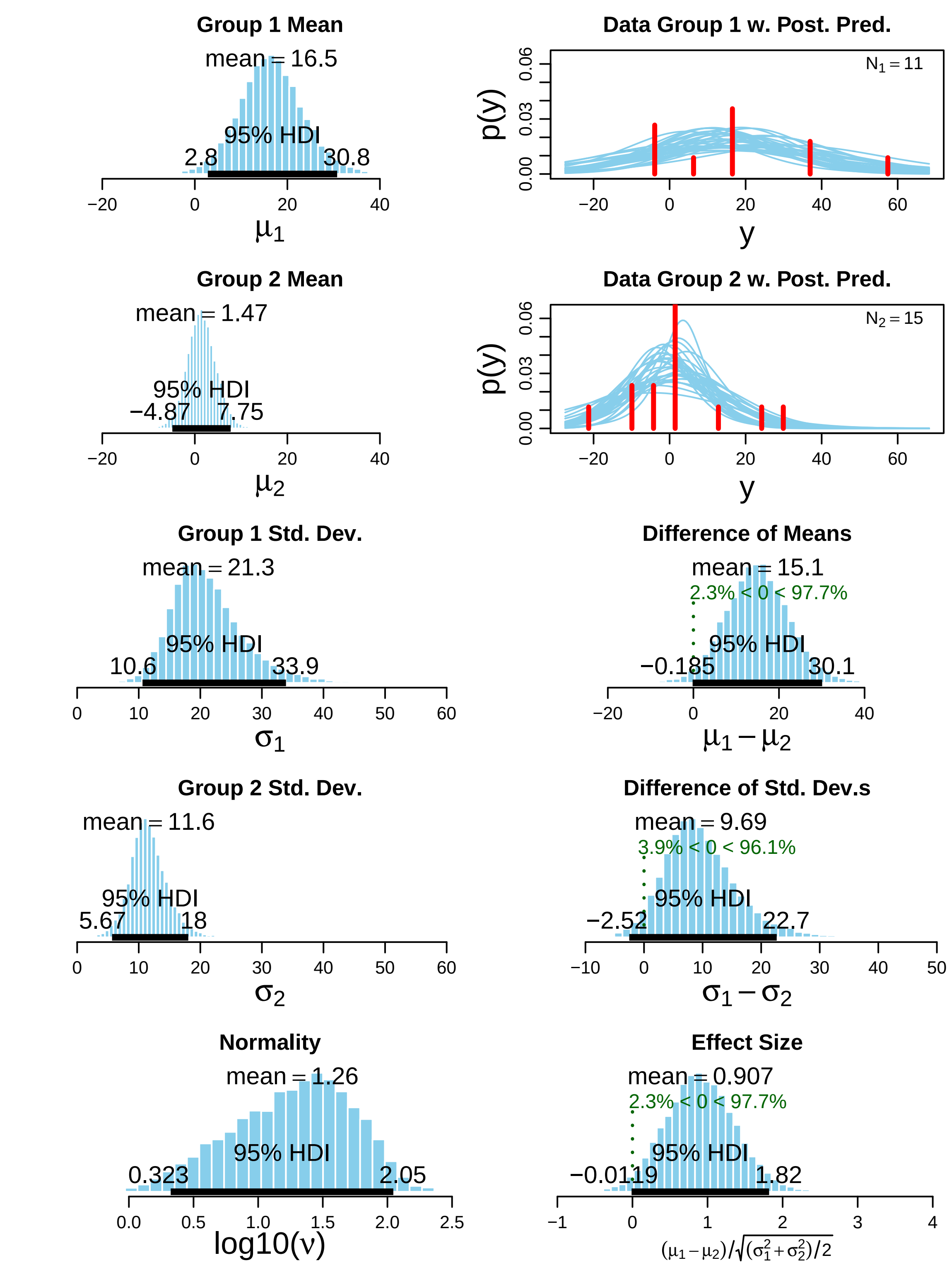
Group 1 Mean
mean = 16.5
95% HDI
2.8
30.8
−20 0 20 40
μ1
Data Group 1 w. Post. Pred.
N1 = 11
p(y)
0.00 0.03 0.06
−20 0 20 40 60
y
Group 2 Mean
mean = 1.47
95% HDI
−4.87
7.75
−20 0 20 40
μ2
Data Group 2 w. Post. Pred.
N2 = 15
p(y)
0.00 0.03 0.06
−20 0 20 40 60
y
Group 1 Std. Dev.
mean = 21.3
95% HDI
10.6
33.9
0 10 20 30 40 50 60
σ1
Difference of Means
mean = 15.1
2.3% < 0 < 97.7%
95% HDI
−0.185
30.1
−20 0 20 40
μ1 − μ2
Group 2 Std. Dev.
mean = 11.6
95% HDI
5.67
18
0 10 20 30 40 50 60
σ2
Difference of Std. Dev.s
mean = 9.69
3.9% < 0 < 96.1%
95% HDI
−2.52
22.7
−10 0 10 20 30 40 50
σ1 − σ2
Normality
mean = 1.26
95% HDI
0.323
2.05
0.0 0.5 1.0 1.5 2.0 2.5
log10(ν)
Effect Size
mean = 0.907
2.3% < 0 < 97.7%
95% HDI
−0.0119
1.82
−1 0 1 2 3 4
(μ1 − μ2)/√((σ1² + σ2²)/2)

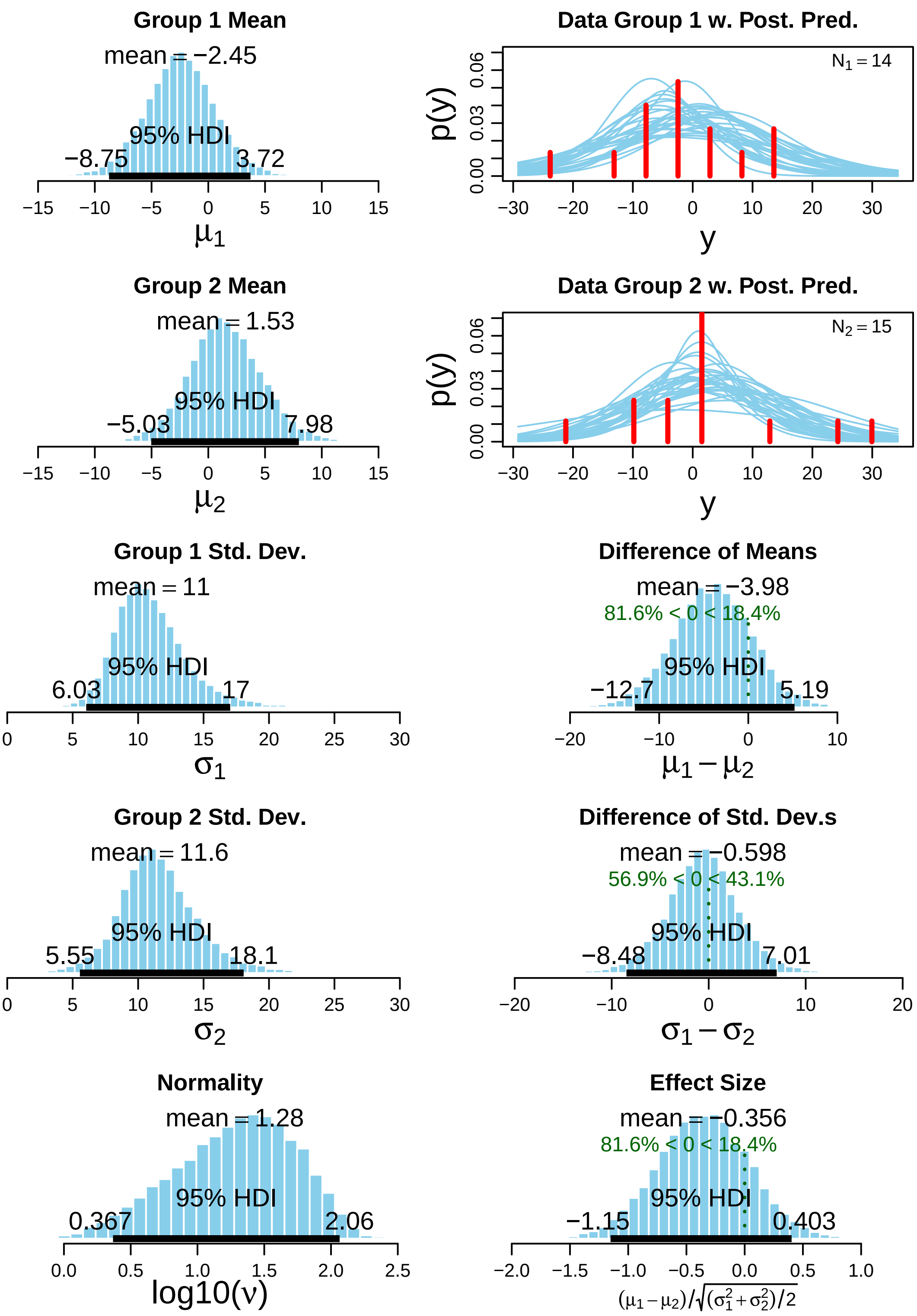
Group 1 Mean
mean = −2.45
95% HDI
−8.75
3.72
−15 −10 −5 0 5 10 15
μ1
Data Group 1 w. Post. Pred.
N1 = 14
p(y)
0.00 0.03 0.06
−30 −20 −10 0 10 20 30
y
Group 2 Mean
mean = 1.53
95% HDI
−5.03
7.98
−15 −10 −5 0 5 10 15
μ2
Data Group 2 w. Post. Pred.
N2 = 15
p(y)
0.00 0.03 0.06
−30 −20 −10 0 10 20 30
y
Group 1 Std. Dev.
mean = 11
95% HDI
6.03
17
0 5 10 15 20 25 30
σ1
Difference of Means
mean = −3.98
81.6% < 0 < 18.4%
95% HDI
−12.7
5.19
−20 −10 0 10
μ1 − μ2
Group 2 Std. Dev.
mean = 11.6
95% HDI
5.55
18.1
0 5 10 15 20 25 30
σ2
Difference of Std. Dev.s
mean = −0.598
56.9% < 0 < 43.1%
95% HDI
−8.48
7.01
−20 −10 0 10 20
σ1 − σ2
Normality
mean = 1.28
95% HDI
0.367
2.06
0.0 0.5 1.0 1.5 2.0 2.5
log10(ν)
Effect Size
mean = −0.356
81.6% < 0 < 18.4%
95% HDI
−1.15
0.403
−2.0 −1.5 −1.0 −0.5 0.0 0.5 1.0
(μ1 − μ2)/√((σ1² + σ2²)/2)

Supplementary Figure S1. Bayesian analyses of the discrimination index carried out for paired comparison between groups Fish were observed for 15 min on day 14 and the time spent near the objects were considered as memorization (object A_1 vs. A_2), then fish were observed again for 15 min on the next day and the time spent near the objects were considered exploration (A_3 vs. B). An exploration index was calculated for the memorization phase and a discrimination index for the discrimination phase. On each comparison of a pair of groups, right side exhibits posterior distribution of the Bayesian analysis: comparison between the exploration time of objects A_1 vs. A_2 and A_3 vs. B for the five groups tested. Green dashed lines (central dashed line) indicate the comparison value = 0 and red dashed lines (lateral dashed lines) indicate the ROPE ± 5. 95 % HDI interval is marked by the black bar on the floor of the distribution. For further details and statistical analyses, see the Results section.
